# A longitudinal study of the 5xFAD mouse retina delineates Amyloid beta (Aβ)-mediated retinal pathology from age-related changes

**DOI:** 10.1186/s13195-025-01784-w

**Published:** 2025-06-19

**Authors:** Savannah A. Lynn, Sudha Priya Soundara Pandi, Aida Sanchez-Bretano, Anna-Marie Muir, Lidia Parker, David S. Chatelet, Tutte Newall, Jennifer A. Scott, Eloise Keeling, Neil R. Smyth, Jay E. Self, Andrew J. Lotery, Helena Lee, J. Arjuna Ratnayaka

**Affiliations:** 1 Clinical and Experimental Sciences, Faculty of Medicine, University of Southampton, Tremona Road, Southampton SO16 6YD, MP806 UK; 2 Biomedical Imaging Unit, University of Southampton, Tremona Road MP12, Southampton, SO16 6YD UK; 3 School of Biological Sciences, University of Southampton, Southampton General Hospital, Southampton, SO16 6YD UK; 4 Eye Unit, University Hospital Southampton NHS Foundation Trust, Southampton, SO16 6YD UK

**Keywords:** 5xFAD, Retina, Mouse model, Amyloid beta (Aβ), Age, Retinopathy, Age-related macular degeneration (AMD)

## Abstract

**Background:**

Age-related macular degeneration (AMD) is the commonest cause of irreversible blindness in developed societies. AMD coincides with advanced age to which genetic and lifestyle factors contribute additional risks. High levels of the Alzheimer’s-linked Amyloid beta (Aβ) proteins are correlated with aged/AMD retinas. To delineate the role of Aβ in retinopathy from age-related changes, we used transgenic 5xFAD mice in a longitudinal study to recapitulate the aged/AMD Aβ-burden of the human retina.

**Methods:**

Mice were genotyped to exclude the retinal degeneration alleles *Pde6b*^*rd1*^, *Pde6brd8*, *Agouti*, *Tyr* and *Oca2*. Retinas of 5xFAD and wildtype littermates (97 males/females in total) were longitudinally assessed until 15 months using non-invasive retinal scans: multi-focal electroretinography, optokinetic tracking, optical coherence tomography (OCT), colour fundus photography and fluorescein angiography. Mice were killed at 4, 8 and 15 months, and eyes enucleated for analyses by light, confocal and electron microscopy.

**Results:**

Age-related changes included a gradual decline of retinal activity in all mice. Subretinal/drusen-like deposits increased with age, but, like retinal vessel morphology and vessel integrity, showed no differences between cohorts. Diminished PSD95 levels indicated impaired photoreceptor-bipolar connectivity which correlated with age. Ultrastructural imaging showed increased electron-dense granules and undigested outer segments within retinal pigment epithelial cells with age. 5xFAD pathology included significant weight reduction vs. wildtype/littermates, which were pronounced in females. 8 month old 5xFAD mice had diminished A and B waves, though the age-related decline in wildtype mice abolished these subsequently. Visual acuity/function was also reduced in 14 month 5xFAD eyes. OCT revealed thickened photoreceptor nuclei and inner segments in 8 month 5xFAD retinae. Scrutiny of chorioretinal tissues revealed diminished photoreceptor nuclei in 4 month 5xFAD eyes, though differences were abolished as both cohorts aged. From 8 months onwards, 5xFAD mice possessed fewer bipolar cell nuclei.

**Conclusions:**

Chronic Aβ exposure led to the earlier development of retinopathy-linked features, the identification of which advances our understanding of how Aβ contributes to multifaceted retinopathies. These were distinguishable from wider age-related changes and non-specific influences of retinal degeneration alleles in 5xFAD mice. Longitudinal analyses revealed sex and age-related limitations and important 3Rs considerations for future studies using 5xFAD mice.

**Supplementary Information:**

The online version contains supplementary material available at 10.1186/s13195-025-01784-w

## Introduction

Age-related macular degeneration (AMD) is the commonest cause of blindness in developed societies, where irreversible damage to the macula, the central part of the retina responsible for activities such as reading, driving and recognising faces, leads to central vision loss. Advanced age is the most notable risk association for AMD, to which genetic and lifestyle factors contribute varying levels of additional risks. This complex aetiology results in a heterogenous population of AMD patients with different disease onset times and rates of progression, but culminating in two well-defined late stage phenotypes termed geographic atrophy (GA, also called dry AMD) or neovascular (wet) AMD. The GA and neovascular phenotypes appear to be distributed equally amongst patients with end-stage AMD [1], though this may differ between certain ethnic groups and/or populations. The prevalence of late AMD in the United Kingdom amongst ≥ 50 years is 2.4%, which increases to 4.8% in people aged 65 years or more and 12.2% in those aged ≥ 80 years [1]. The worldwide prevalence of AMD, which accounts for 8.7% of all blindness globally, was estimated at 196 million patients in 2020 and projected to affect 288 million individuals by 2040 [2]. Neovascular AMD is managed in most cases through repeated intravitreal anti-vascular endothelial growth factor (VEGF) injections. However, despite this, vision deteriorates over time [3]. GA patients currently have no effective treatments whatsoever.

Although significant advances have been made in identifying the genetic landscape of AMD [4], with recent progress made in delineating lifestyle factors, particularly dietary effects [5, 6], risks such as the correlation of Alzheimer’s-linked Amyloid beta (Aβ) proteins in the ageing and AMD retina, remains to be further understood. Histopathological analyses of aged/AMD donor eye tissues show high Aβ levels accumulating in the retina. These pathogenic proteins, the toxic effects of which have been extensively described in the neuropathology of the brain [7], show a preferential pattern of deposition in the human and non-human primate retina. Prominent amongst these retinal Aβ hotspots are drusen, which are subretinal lipid-protein deposits associated with ageing and retinopathy [8]. Aβ assemblies were most frequently correlated with retinas containing moderate to high drusen loads [9]. Further scrutiny revealed Aβ organised in vesicular components within drusen referred to as ‘amyloid vesicles’ [9–12] which were reported in macular and peripheral drusen from donors with and without AMD [10], whilst other studies associated Aβ containing drusen only with donor tissues from AMD patients [13]. Measurement of plasma Aβ levels in circulation [14] as well as in aqueous samples [15] showed an association with late AMD stages, which collectively describe an age-associated Aβ-burden in the retina that is linked with poorer outcomes for AMD patients [8]. Recent discoveries reporting increased retinal Aβ levels correlated with worsening mild cognitive impairment (MCI) and Alzheimer’s disease (AD) [16] as well as cerebral amyloid angiopathy [17], also link retinal Aβ with well-characterised neuropathology in the brain.

Researchers have developed AD rodent models, particularly transgenic mice, many of which exhibit accelerated and high levels of Aβ production in the brain, that have since been exploited to investigate Aβ pathology in whole retinas. Amongst these models, the 5xFAD (familial Alzheimer’s disease) mice rapidly generate high levels of the most pathogenic and aggregate-prone Aβ_1–42_ form, driven by the presence of 3 mutations in the human knock-in amyloid precursor protein (*APP*) and 2 mutations in the human presenilin-1 (*PSEN1*) genes that are linked with AD [18]. Of relevance to this study is that 5xFAD mice also generate some of the highest levels of ocular Aβ levels amongst AD rodent models [19], making them an ideal tool to investigate Aβ effects in the ageing retina. However, a major consideration omitted in studies is the importance of excluding *Pde6b*^*RD1*^, *Pde6b*^*RD8*^, *Agouti*, *Tyr* and *Oca2* genes, which are associated with retinal degeneration, that may be carried in this transgenic model. Here, we used 5xFAD mice to understand how the age and AMD-associated retinal Aβ-burden can affect the development of retinopathy. By backcrossing 5xFAD (Tg6799) with C57BL6/J mice, which presents an improved model for retinal studies and by confirming the exclusion of the aforementioned retinal degeneration alleles, our findings were able to unambiguously delineate Aβ effects in the retina from other age-related retinal changes. By using a series of non-invasive retinal assessments that are routinely used in AMD patients across the lifecourse of 5xFAD mice, our study obtained insights into dynamic Aβ effects in living retinas, where non-transgenic wildtype (Wt) littermates acted as controls. We also investigated 5xFAD mice retinas for up to 15 months, a substantially long period for mice with an advanced neuropathological phenotype [18], thus extending the scope of studies to encompass old age and the likely limit of the 5xFAD model. Light and confocal microscopy as well as ultrastructural scrutiny with electron microscopy of retinas from enucleated eyes at 4, 8 and 14–15 months provided a histopathological snapshot and details of Aβ-mediated retinal pathology. Our combinational approach therefore provided a longitudinal map of Aβ driven pathology vs. wider age-related alterations in the retina across the lifecourse, offering insights into this important aspect of ageing and its role in retinopathy.

## Methods and materials

### Animal housing and husbandry

All experiments were performed in the light stage of the light–dark cycle (8:00–16:00h) on test naive male and female C57BL6/J 5xFAD mice (MMRRC Stock:34,848-JAX) and control C57BL6/J littermates bred and maintained within the Biomedical Research Facility (BRF) at the University of Southampton. Studies were overseen by the institutions’ Animal Welfare and Ethical Review Board (AWERB) and were consistent with the Association for Research in Vision and Ophthalmology (ARVO) statement for the Use of Animals in Ophthalmic procedures and the UK Animal (Scientific Procedures) Act of 1986. The study complied with the ARRIVE guidelines, with work undertaken under a UK Home Office project licence. Animals were kept under similar conditions to those described previously [20]. Briefly, mice were allowed access to standard laboratory chow and water ad libitum, and housed in conventional cages containing environmental enrichment and Lignocel 2/2 bedding (IPS Ltd., London, UK) whilst being maintained on a 12h/12h light–dark cycle at 19—24°C with a maximum of 10 mice per cage. Body weight was monitored throughout the lifespan of mice as an additional measure of welfare, where studies were terminated if a change > 10% was observed over 3 days or if animals dropped > 20% maximal (typically 8 months) body weight throughout the experimental duration (Supplementary Table S1). To this end, wet standard laboratory chow was also given to animals at later timepoints (12—14 months). Female mice (*n* = 51) were used for longitudinal studies requiring repeated anaesthesia (electroretinography and optical coherence tomography) and males (*n* = 45) for single anaesthetic procedures (colour fundus photography and fluorescein angiography) to avoid anticipated complications related to repeated anaesthesia in ageing mice. This approach was adopted as preliminary studies resulted in obstructive uropathy and premature death following repeated dosage of ketamine/dexmedetomidine hydrochloride in male 5xFAD mice. Both genders (n = 56) were used for longitudinal behavioural experiments (optometry). Further details regarding the exact number of mice (experimental unit) within groups and timepoints are described in the corresponding figure legend for each experiment. A total of *n* = 97 mice were used for the study. Statistical power was determined via priori sample size calculation using preliminary electroretinography data (8 months) from pilot experiments and online software (https://www.stat.ubc.ca/~rollin/stats/ssize/n2.html). The analysis method was two-tailed using a significance threshold of 0.05%, experimental power of 80%, standard deviation of 63.19 and means of 278.3 and 224.3. A total of 22 mice were considered necessary for each group. All data acquisition and analyses were performed blind to the genotype (5xFAD or Wt littermates) of the experimental animal.

### Genotyping

Stock male 5xFAD mice were purchased from The Jackson Laboratory (MMRRC Stock: 34,848-JAX) after being confirmed to be comparable to a C57BL6/J reference sample genome for *Pde6b*^*rd1*^, *Pde6brd8*, *Agouti* and *Tyr_c2j*. Upon receipt, mice were screened for additional retinal mutations associated with SJL lineage prior to commencing breeding and were found to be wild type for *Agouti*, *Tyr* and *Oca2* (Supplementary Figure S1). All subsequent 5xFAD progeny were genotyped according to the following method to determine their genetic status. Briefly, DNA was extracted from ear biopsies taken from mice aged > 21 days according to the hot sodium hydroxide and tris (HotSHOT) method [21], where 75µl of 25mM NaOH/0.2mM EDTA was added to samples and incubated at 98 °C for 2 h in a MJ Research Tetrad 2 Thermal Cycler (Bio-Rad, UK) before being neutralised with 75µl of 40mM Tris–HCl and spun at 4000 rpm for 3 min in a Micro Laboratory Centrifuge. PCR reactions comprising 13.42µl H_2_O, 0.6µl MgCl_2_, 0.4µl dNTPs, 1µl 10µM forward primer, 1µl 10µM reverse primer, 0.08µlTaq polymerase (5U/µl), 2µl 10 × PCR buffer (200mMTris-HCl pH 8.4, 500mM KCl) and 0.5µl of extracted DNA were subsequently set up and run in a Thermal Cycler according to the conditions described in (Supplementary Table S2). Agarose gel electrophoresis (2% gel, 45 min,140V) was then carried out on amplified PCR products alongside a 100bp ladder to determine mouse genotypes, where heterozygous 5xFAD transgenic mice were observed to contain 129bp and 216bp bands with Wt littermates showing only a single 216bp band. For initial screening of stock males for *Oca2*, *Tyr* and *Agouti* genotypes, the reaction consisted of 12.9µl H_2_O, 4µl HF Buffer, 0.4l dNTPs, 1µl 10µM forward primer, 1µl 10µM reverse primer and 0.2µl Phusion with 0.5µl of extracted DNA subject to PCR as described in Supplementary Table S2. The reaction products were resolved on a 1% agarose gel at 120 V for 40 min. Restriction digests with BstEII and HypCH4III were subsequently performed for *Oca2* and *Tyr* respectively and were resolved on a 1.5% agarose gel at 70 V for 140 min to confirm genotypes. The expected band sizes are described in Supplementary Table S2.

### Animal preparation and recovery

At the time of experimentation, mice received an intraperitoneal (IP) injection of ketamine (75mg/kg) (Bayer PLC, Reading, UK) and dexmedetomine hydrochloride (1mg/kg) (Centaur Services, Castle Carry, UK) and were placed in the dark until motor function ceased. The use of ketamine promotes dilation of the pupil, making it an optimal anaesthetic for conducting functional and visual assessments of the eye. A small amount of 2.5% w/v phenylephrine hydrochloride (Chauvin Pharmaceuticals Ltd., London, UK) followed by 1% w/v Tropicamide (Chauvin Pharmaceuticals Ltd., London, UK) was then applied to eyes for 2 min each to facilitate pupillary dilation, after which ocular hydration was maintained throughout procedures via repeated application of Viscotears (Alcon, Farnborough, UK). In all procedures, the order of assessment was allocated using an online randomiser tool (https://www.random.org/lists). Animals were recovered via intraperitoneal injection with 2mg/kg antipamezole hydrochloride (Centaur Services, Castle Carry UK) and were maintained on a heat pad.

### Electroretinography (ERG)

Prior to acquiring ERG recordings, mice were transferred to red Leddy IVC cages (Tecnhiplast, UK) and dark adapted in a controlled IVC air flow system for 12 h before being prepared as described in the previous section. Once under anaesthesia, animals were secured on a heated platform connected to the ground electrode (tail), reference electrode (head) and corneal electrode (gold-plated objective lens) following lubrication of eyes with Viscotears (Alcon, Farnborough, UK). Multi-focal scotopic electroretinograms (ERGs) were then obtained using the Micron III Retinal Imaging System and Second Generation Image-Guided ERG attachment (PhoenixResearch Labs, Pleasanton, CA, USA) as two sweeps via stimulation with 1.5mm diameter, 6.8 cd-s/m2 white LED light for 1ms with a 2 min interval. As before, OS measurements were conducted first for experimental consistency. Average A and B wave amplitudes along with corresponding implicit times T(A) and T(B) were calculated in the V3 Phoenix LabScribe ERG software suite (Phoenix Research Labs, Pleasanton, CA, USA) and were taken forward for statistical analyses. A and B-wave amplitudes were calculated as the measurement from baseline to the A wave trough and the A wave trough to the B wave peak, respectively. The implicit time was calculated as the time interval between stimulus onset and the wave peak for both the A and B waves. To minimise variations in readings, the room temperature was pre-warmed and maintained at 27 °C throughout the procedure. Similarly, all equipment was placed inside a 6-panel aluminium copper mesh Faraday cage (Micro Control Instruments Ltd., Framfield, UK*)* to prevent external electrical interference.

### Optokinetic tracking (OKT) response

The optokinetic tracking (OKT) response was assessed as a measure of visual acuity and function using the OptoMotry System (CerebralMechanics Inc., Canada) as described previously [22–24]. Briefly, mice underwent an initial training session to familiarise them with the machine and were habituated for approximately 5 min prior to subsequent experimentation. During the test procedure, the animal was placed on a central pedestal in an experimental arena consisting of 4 connected liquid crystal display (LCD) monitors with a motioned black and white sine-wave grating drawn in 3D space which rotated at a speed of 12deg/sec around the centre platform. The spatial frequency of the grating was then systematically increased (0.03c/d) at 100% contrast in the OptoMorty HD software (CerebralMechanics Inc., Canada) and visually evoked reflexive head and neck movements monitored by an assessor from a top-down camera to determine the photopic spatial frequency thresholds at which the mice ceased to track gratings. A positive tracking response was defined as a smooth directional head movement in line with the displayed sine-wave grating rotation. This process was performed for gratings rotating randomly in either the clockwise (CW) and counter-clockwise (CCW) direction, or up and down direction with the OptoMotry HD software suite (CerebralMechanics Inc., Canada) recording outputs. Mice were allowed to move freely whilst exposed to visual stimuli and were returned to the pedestal using the tube handling method in the event they left the pedestal.

### Optical Coherence Tomography (OCT)

Optical coherence tomography was performed immediately following ERG recordings using the Leica Envisu R2200 VHR SDOIS Mouse Imaging system (BioptigenInc., Durham, NC, USA) as described previously [20]. Briefly, mice were wrapped in surgical gauze and positioned comfortably within a stereotactic rotational cassette with their heads aligned on a bite bar in an animal imaging mount. Mouse eyes were cleansed with Systane lubricant eye drops (Alcon, Camberley, UK) and surgical spears, with eyes positioned to facilitate alignment with the retina lens. Scans were then centred around the optic nerve head (ONH) using the multiaxial rodent alignment stage apparatus and *en-face* fundus and retinal cross-sectional previews displayed in the InVivoVue Clinic software (Bioptigen Inc., Durham, NC, USA). Volumetric scans (1.4mm^2^) consisting of 100 B scans and 1000 A scans were acquired through a 50° field of view. Scans were segmented in the InVivoVue 2.4 Diver automated analysis software (Leica Microsystems, Milton Keynes, UK) using the semi-automated segmentation function for the Retinal Nerve Fiber Layer (RNFL), Inner Plexiform Layer (IPL), Inner Nuclear Layer (INL), Outer Plexiform Layer (OPL), Outer Nuclear Layer (ONL), Inner Segments (IS), Outer Segments (OS) and the Retinal Pigment Epithelium (RPE). Segmentation was performed at 24 retinal locations centred around the ONH which were within a 600µm radius and spaced on a grid with 238µm increments. The term semi-automated segmentation is used as retinal layer borders were first marked manually at each of the 24 locations following placement of the analysis grid around the ONH, prior to the programme then calculating the thickness in microns for each of the individual retinal layers as described before [25]. Average thicknesses of component retinal layers as well as total (RNFL—RPE), inner (RNFL—INL), middle (OPL—ONL) and outer (IS—RPE) retinal thicknesses were calculated from both eyes for the purpose of statistical analyses.

### Funduscopy

Colour fundus photographs (CFP) of male C57BL6J/5xFAD and C57BL6/J mouse retinae were obtained as described previously [20] using the Micron III Retinal Imaging System (Phoenix Research Labs, Pleasanton, CA, USA) one week prior to experimental endpoints (4, 8 and 15 months). Briefly, mice were secured on a heated imaging platform allowing easy manoeuvrability of their hind legs. Viscotears (Alcon, Farnborough, UK) was applied to the eye being imaged and the cornea aligned with the microscope lens. Eyes were positioned with the optic nerve at the centre of the field of view in the Micron III Retinal Imaging Microscope Software Suite (Phoenix Research Labs, Pleasanton, CA, USA) and images acquired using the brightfield imaging modality (450—650nm). In all instances, *Oculus Sinister* was imaged first to ensure consistency, with fluorescein angiography performed immediately after CFP images were collected for methodological uniformity.

### Fluorescein Angiography (FFA)

In order to maintain ocular alignment, fluorescein angiography was performed immediately following CFP using the Micron III Retinal Imaging System and Software Suite (Phoenix Research Labs, Pleasanton, CA, USA). Mice received an IP injection with 75µl of 2% w/v fluorescein sodium in physiological saline (Bausch & Lomb, NY, USA) by careful elevation of the hind leg and controlled continuous depression of the syringe. FFA images were then obtained immediately using the FFA imaging modality (490nm barrier filter). The development of the fluorescence signal was monitored over a period of 5 min with 24-bit RGB TIFF files acquired sequentially every 20 s until vascular saturation (OD). However, FFA images of saturated vessels only were acquired for OS due to the inability to monitor temporal FFA accumulation bilaterally. FFA image analysis to determine retinal vessel morphology and the avascular region was performed in Image J [26] (NIH, USA) or MATLAB (MathWorks, MA, USA) using the open source Quant BV Code [27].

### Histological analyses of chorioretinal mouse tissues

Animals were culled via terminal anaesthesia with 0.2ml Pentobarbital (MWI Animal Health, UK) and transcardial perfusion with 0.1% NaCl and Heparin Sodium (5 Units/ml). Enucleated eyes were immediately transferred to foil cups containing optimal cutting medium to be snap frozen inside a glass dish filled with isopentane placed on dry ice. Each retinal sections of size 6 μm were then obtained using a Leica CM1860 W Cryostat (Leica Microsystems, UK) and collected consecutively on 15 SuperFrost™ Plus glass microscope slides (Fisher Scientific, UK) with approximately 12 sections per slide. Prior to staining, slides were dried in a Cole-Palmer Stuart SI60D forced air incubator (Fisher Scientific, UK) at 37 °C for 1 h and washed thrice in 1 × PBS for 10 min each to remove the optimal cutting medium surrounding the tissue.

### Toluidine blue staining

Toluidine blue staining was performed on semi-thin (0.5µm) sections embedded in EM fixative, which were collected in a droplet of dH_2_0 on SuperFrost™ glass microscope slides and dried on a hot plate set to 100 °C for approximately 15 min. A solution of 1% toluidine blue in 1% borax (Electron Technology, Stanstead, UK) was then applied to sections until the edges of the solution turned sea green (~ 30 s) and slides gently rinsed in ddH_2_0 to remove residual stain. Slides containing sections were subsequently mounted with DPX mounting medium and imaged using a LM Nikon Eclipse 80i DIC fitted with a Nikon DN100 camera. Quantification of histological features was performed in Image J [26] (NIH, USA) using the Cell Counter plugin.

### Confocal immunofluorescence microscopy and image analysis

Fresh frozen chorioretinal sections were fixed in 4% PFA for 10 min, washed three times in 1xPBS and blocked in 10% NGS in 0.3% PBST for 1 h at room temperature before being incubated with anti-PSD95 (2507s, 1:100, RRID: AB_561221, Cell Signalling Technologies, UK) prepared in blocking buffer overnight. The following day, sections were washed thrice in 1xPBS, incubated with goat anti-rabbit 488 (A11070, 1:200, RRID:AB_142134, Thermo Fisher Scientific, UK) prepared in 1 × PBS for 1 h at room temperature, washed an additional three times in PBS and incubated with 1mg/mL DAPI (D9542, Sigma Aldrich, UK) for 10 min. Sections then were subject to a final wash step in 1xPBS prior to being mounted with Mowiol mounting medium on a glass coverslip. Retinal sections were imaged on an Olympus VS110 fluorescent slide scanner (Olympus, UK) at (× 20 magnification). Image analysis was carried out in anonymised chorioretinal sections using Fiji/ImageJ software (version 1.54f) where n ≥ 3 images were collected from each mouse eye. Briefly, a green pseudo colour overlay was applied to 8-bit images of PSD95 labelling. The bipolar-photoreceptor interface identified by PSD95 staining was delineated by drawing a region of interest (ROI) with a 300 pixel margin. That ROI was then straightened using the ‘straighten’ function from Fiji and non-specific background staining eliminated by a mask. Finally, a threshold based on the extent of labelling in 8 month old Wt mice was applied to all images to quantify the mean intensity of the PDS95 label. A custom-made Fiji macro was written to automate this process and is shown in the supplementary information.

### Transmission electron microscopy (TEM)

For TEM, animals were culled via terminal anaesthesia with 0.2ml Pentobarbital (MWI Animal Health, UK) and transcardial perfusion with 0.1% NaCl and Heparin Sodium (5 Units/ml). Mice were enucleated and eyes transferred to primary fixative comprising 3% glutaraldehyde and 4% formaldehyde in 0.1M PIPES (pH 7.2) buffer for a minimum of 3 days at 4 °C before the anterior segment was removed under a Leica DM100 LED dissection microscope (Leica Microsystems, UK). The eyes were then returned to the primary fixative to allow further penetration of tissues. Eyes were subsequently rinsed twice in 0.1M PIPES for 10 min and post fixed in 1% osmium tetroxide in 0.1M PIPES for 1 h. Following osmification, an additional two 10 min rinses in 0.1M PIPES were performed. Eyes were washed briefly in ddH_2_O, block stained in 2% uranyl acetate (aqueous) for 20 min and then dehydrated by passing the samples through a battery of ethanol gradients (30%, 50%, 70% and 95%) for 10 min each. This was followed by two 20 min incubations with absolute ethanol (100%) and application of the link reagent acetonitrile for 10 min followed by an overnight incubation in a 1:1 ratio of acetonitrile to Spurr resin (Agar Scientific, Stanstead, UK). Finally, eyes were transferred to Spurr resin for 6 h and subsequently polymerised in fresh resin at 60 °C for 24 h. Semi-thin microtome sections (0.5µm) were cut using a Reichert Ultracut E ultramicrotome (Leica Microsystems, UK) and stained with 1% toluidine blue in 1% borax (Electron Technology, Stanstead, UK) for examination prior to cutting silver/gold ultrathin sections. These ultrathin sections (100nm) were collected on 200 mesh copper grids and stained with Reynold’s Lead stain prior to imaging using a Hitachi HT77000 transmission electron microscope (Hitachi, Germany). A minimum of three micrographs were acquired per grid across n ≥ 3 eyes per time point (4, 8 and 15 months) and quantification performed in Image J (NIH, USA). All procedures were carried out at room temperature unless otherwise stated. For quantification purposes, the parameter under assessment was quantified in at least 3 images from each statistical unit and the average taken for statistical analysis. Quantification was performed using the count and measure functions in Image J [26].

### Statistical analyses

Statistical analysis was performed in GraphPad Prism Software version 10.1.0 (264) for macOS (GraphPad, San Diego, CA) following guidance from a medical statistician. Data was initially assessed for Gaussian distribution and outliers using ROUT test (Q = 1%) followed either by repeated measures two-way ANOVA (mixed model) with Bonferroni correction, two-tailed unpaired t-test or Shapiro–Wilk test with statistical significance denoted as * p ≤ 0.05, ** p ≤ 0.01, *** p ≤ 0.001, **** p ≤ 0.0001. Data is shown as means ± SEM (standard error of the mean) with the experimental unit as the individual animal. Recorded measurements correspond to an average value taken from both eyes in the case of CFP, OCT, ERG and FFA analyses.

## Results

### Genotypic characterisation of 5xFAD mice

5xFAD mice were initially created on a C57BL6/J x SJL/J hybrid background. However, the SJL/J strain carries several mutant alleles that are associated with visual abnormalities including *Pde6b*^*RD1*^, *Pde6b*^*RD8*^, *Agouti*, *Tyr* and *Oca2* genes. As these are passed on in an autosomal recessive pattern, some animals may be homozygous for these alleles which could consequently affect any visual assessments in the progeny [28, 29]. Two strains of 5xFAD mice are available via The Mutant Mouse Resource & Research Centre (MMRRC); #034848-JAX and #034840-JAX, of which the latter is maintained by backcrossing transgenic animals to a B6SJLF1 hybrid, resulting in progeny that may be heterozygous, homozygous or Wt for these alleles. Concerningly, several studies that investigate retinal pathology have utilised this model without any prior screening or only at best with partial screening. By contrast, the MMRRC 034848-JAX strain was produced by backcrossing Tg6799 to C57BL6/J mice, which presents an improved model for retinal studies as it is known to lack *Pde6b*^*RD1*^ and is supplied with a guarantee that mice are Wt for *Pde6b*^*RD8*^, *Agouti* and *Tyr_c2j* (https://www.jax.org/strain/008730), and hence our choice for the present study. Upon receipt, 5xFAD stock males were also screened for *Oca2* to exclude the presence of all retinal degeneration alleles that may influence our results (Supplementary Figure S1).

### Longitudinal analyses of retinal function in 5xFAD mouse eyes

**Fig. 1:**
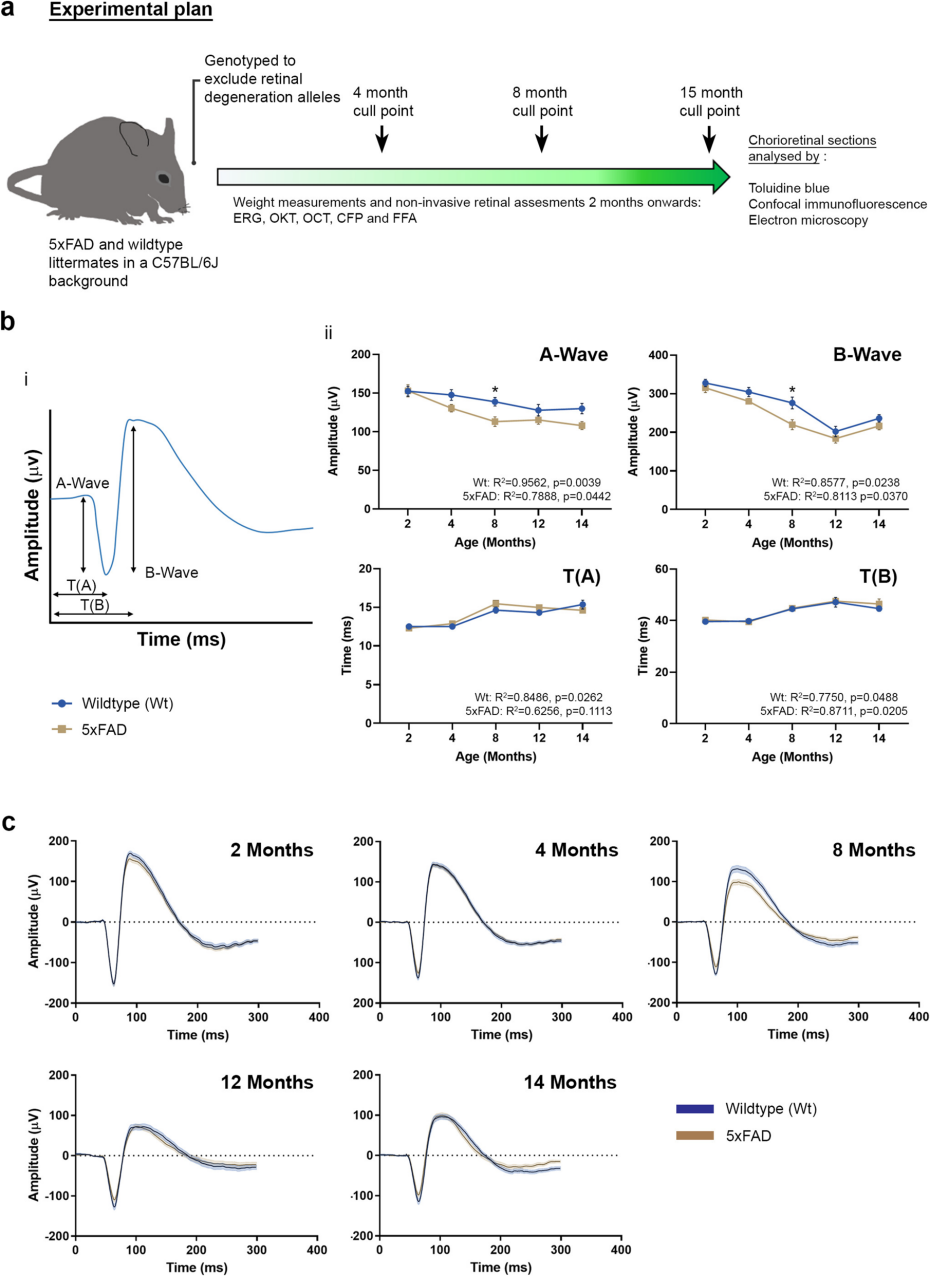
Experimental plan and muti-focal electroretinography (ERG) in 5xFAD and wildtype littermate mice. **a** Experimental set-up showing key milestones and workflow. **b** An overall decrease in the amplitude of A wave and B waves were observed in 5xFAD retinae from 4 months onwards compared to control littermates, suggesting a potential impairment of photoreceptor and inner retinal function, respectively. However, these differences only reached significance at 8 months, after which retinal function was indistinguishable from those in wildtype control mice. Averaged A and B wave implicit times T(A) and T(B) showed no significant differences between the two groups. Statistical comparisons were made using two-way ANOVA (mixed model) with Bonferroni post-hoc test with significance shown as * *p* ≤ 0.05. Data is expressed as means ± SEM (standard error of the mean). **c** Grouped average ERG traces for all timepoints with shaded regions representing SEM. Experimental replicates were as follows. 2 months: Wt (n = 22), 5xFAD (n = 25); 4 months: Wt (n = 22), 5xFAD (n = 30); 8 Months: Wt (*n* = 18), 5xFAD (*n* = 23); 12 Months: Wt (*n* = 13), 5xFAD (*n* = 17) and 14 Months: Wt (*n* = 20), 5xFAD (*n* = 18). Assessed by Pearson’s correlation coefficient

Weights of 5xFAD and Wt littermates may be used as an indicator of overall welfare to inform suitable intervals for longitudinal retinal assessments and associated repeated anaesthesia. Hence, weights of animals were measured throughout the study duration. For detailed 3Rs considerations (replacement, reduction and refinement of animals used in research), values were obtained separately for male and female mice to determine any effects of sex, which is seldom reported (Supplementary Table S1). We observed notable evidence of weight loss as early as 2 months and extending through other timepoints until the 15 month cull-point. Interestingly, female 5xFAD mice lost more weight compared to their Wt counterparts at each timepoint. 5xFAD males also lost weight compared to Wt animals, but the differences were noticeably less severe (Supplementary Figure S2). Both 5xFAD and Wt mice showed increasing vulnerability with old age, which was most noticeable in 5xFAD mice after 12 months. Consequently, to avoid the unnecessarily loss of mice and for improved animal welfare considerations, some non-invasive retinal assessments were limited to selected timepoints with a final measurement taken at 14 months prior to immediate culling or soon after at 15 months (Fig. 1a).

**Fig. 2:**
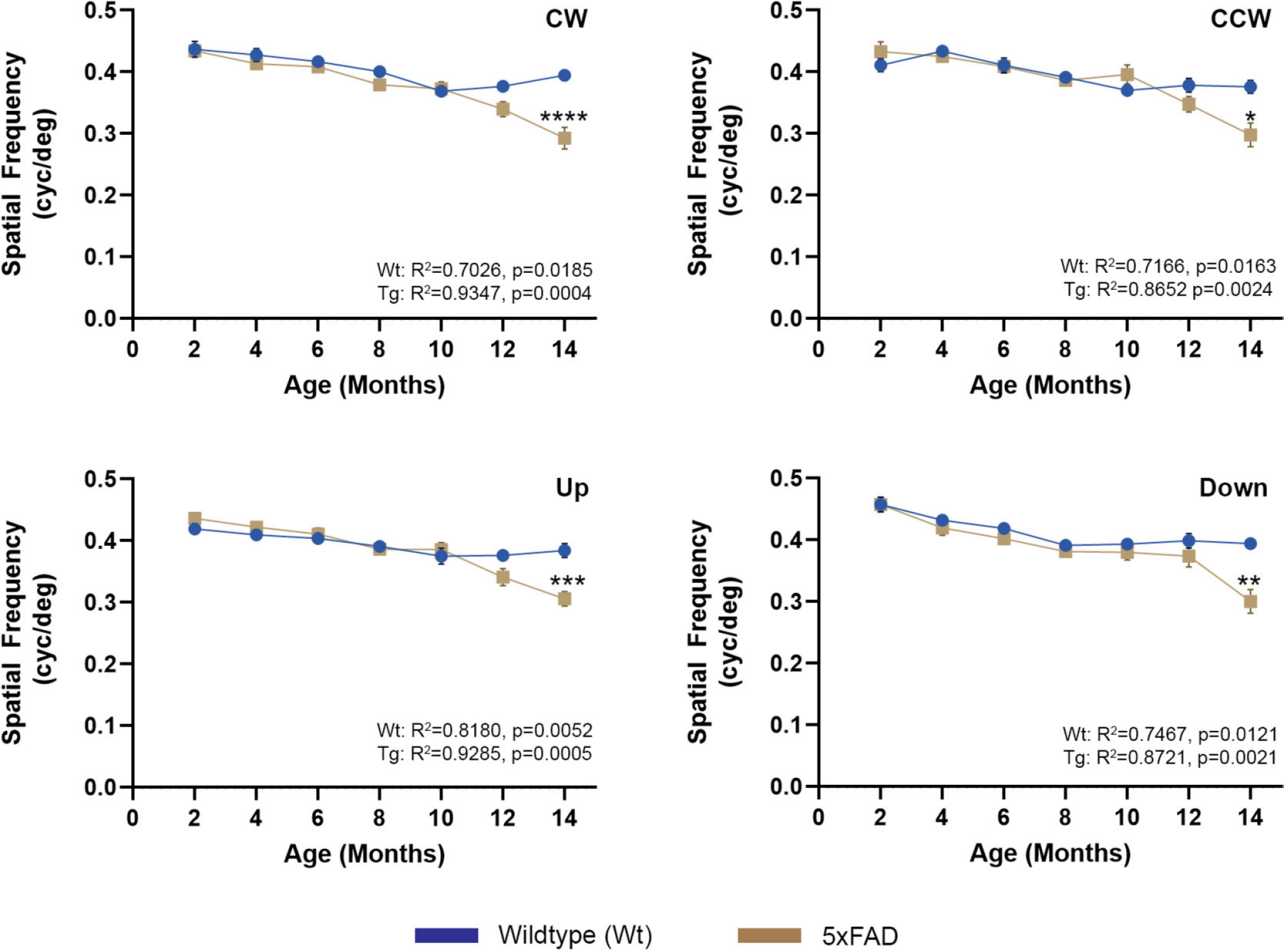
Optokinetic tracking (OKT) responses in 5xFAD and wildtype mice. The optokinetic tracking responses were assessed longitudinally as a measure of visual acuity and function. No significant differences in detectable spatial frequencies were recorded until 14 months, when 5xFAD mice exhibited a decline in clockwise (CW), counter-clockwise (CCW), up and down responses. Data is expressed as means ± SEM (standard error of the mean). Statistical comparisons were made using two-way ANOVA with Bonferroni post-hoc test with significance shown as * *p* ≤ 0.05, ** *p* ≤ 0.01, *** *p* ≤ 0.001, **** *p* ≤ 0.0001. Experimental replicates were as follows. 2 Months: Wt (*n* = 11), 5xFAD (*n* = 14); 4 Months: Wt (*n* = 11), 5xFAD (*n* = 14); 6 Months: Wt (*n* = 11), 5xFAD (*n* = 14); 8 Months: Wt (*n* = 10), 5xFAD (*n* = 14); 10 months: Wt (*n* = 24), 5xFAD (*n* = 28); 12 months: Wt (*n* = 24), 5xFAD (*n* = 27) and 14 months: Wt (*n* = 14), 5xFAD (*n* = 13)

Longitudinal assessments of retinal function were undertaken using dark adapted muti-focal scotopic ERG at 2, 4, 8, 12 and 14 months of age. The amplitude and duration of A and B waves, which report the activity of photoreceptor rods and the inner retina (predominantly ON bipolar cells and to a lesser extent Müller, amacrine and RGCs), respectively, were recorded in 5xFAD mice and in age-matched control littermates (Fig. 1b). From 2 months onwards, 5xFAD mice consistently displayed lower A (5xFAD: R^2^ = 0.7888, *p* = 0.0442 and Wt: R^2^ = 0.9562, *p* = 0.004) and B (5xFAD: R^2^ = 0.8113, *p* = 0.037 and Wt: R^2^ = 0.8577, *p* = 0.024) waves compared to age-matched controls (Fig. 1b upper panels). However, significant differences were only observed by 8 months. Implicit times, which report the time interval between onset of stimulus and peaks for A and B waves (T_(A)_ and T_(B)_) respectively, showed no significant differences between the two cohorts for the duration of these assessments (Fig. 1b lower panels). Additional longitudinal assessments of the mouse visual pathway were carried out using OKT, which provided further insights into the functionality of 5xFAD retinae (Fig. 2). No differences were observed between 5xFAD and age-matched Wt littermate cohorts until 14 months, when the ability to track clockwise motion (5xFAD: R^2^ = 0.9345, *p* = 0.0004 and Wt: R^2^ = 0.7026, *p* = 0.018), counter-clockwise motion (5xFAD: R^2^ = 0.8652, *p* = 0.0024 and Wt: R^2^ = 0.7166, *p* = 0.016), up (5xFAD: R^2^ = 0.9285, *p* = 0.0005 and Wt: R^2^ = 0.8180, *p* = 0.005) or down (5xFAD: R^2^ = 0.8721, *p* = 0.002 and Wt: R^2^ = 0.7467, *p* = 0.012) was significantly diminished in 5xFAD mice (raw data in Supplementary Table S3).

### Longitudinal analyses of retinal structure in 5xFAD mouse eyes

**Fig. 3:**
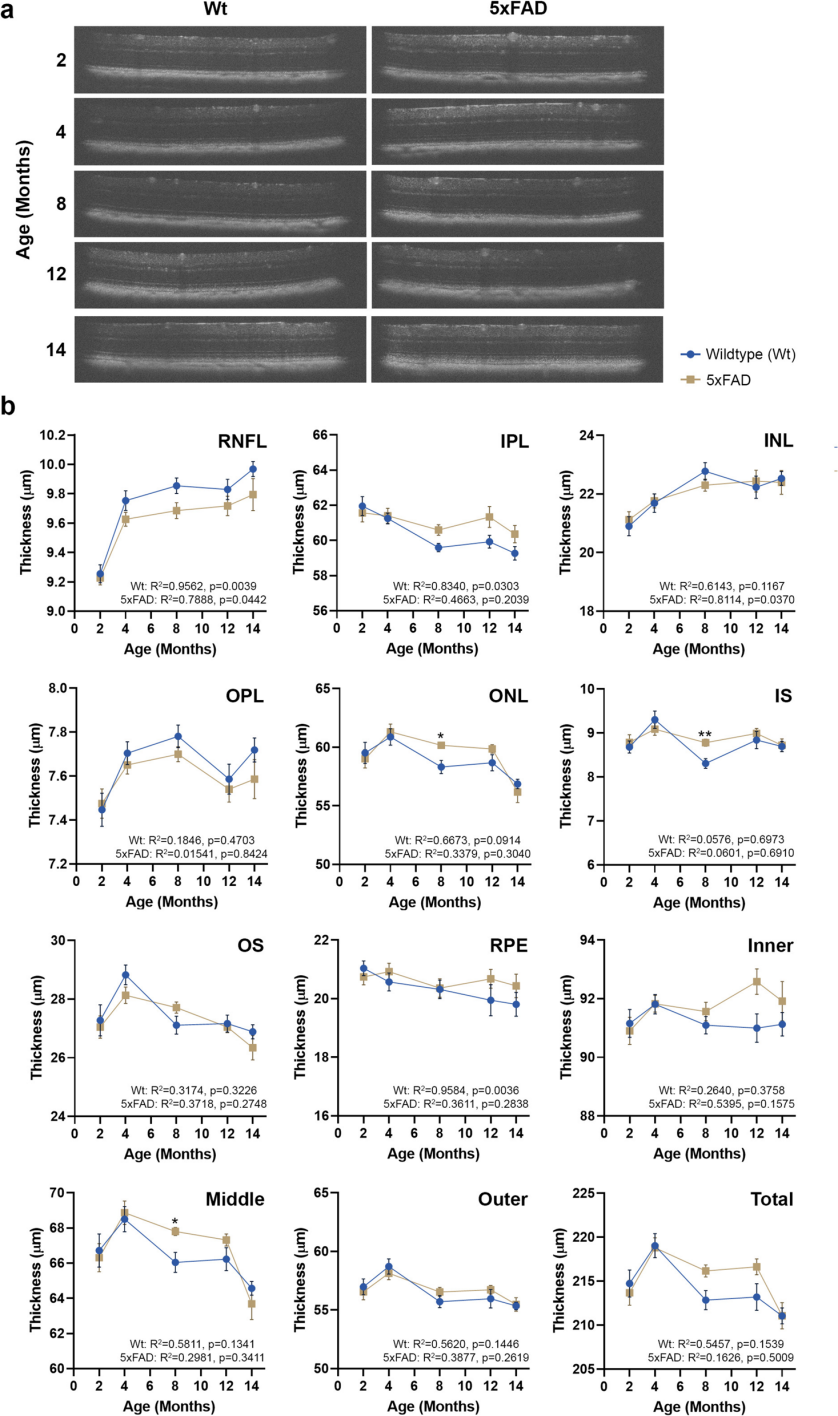
Optical coherence tomography (OCT) measurements of 5xFAD and wildtype mice. **a** OCT scans obtained at 2, 4, 6, 8, 10 12 and 14 months were subject to automatic segmentation using InVivoVue Diver analysis software to assess differences in component retinal layer thicknesses. **b** No differences were recorded in the RNFL, IPL, INL, OPL, OS or RPE layers. However, differences between 5xFAD and wildtype littermate retinae were recorded in the ONL and IS layers (photoreceptors) at 8 months. Measurement of the inner (RNFL-INL), middle (OPL-ONL) and outer (IS-RPE) retinal thicknesses showed changes in the middle retina at 8 months between the two cohorts, which was not evident when summed as the total retinal thickness. Data is expressed as means ± SEM (standard error of the mean). Statistical comparisons were made using a two-way ANOVA with Bonferroni’s post-hoc test with statistical significance shown as * *p* ≤ 0.05 and ** *p* ≤ 0.01. Experimental replicates were as follows. 2 months: Wt (*n* = 22), 5xFAD (*n* = 25); 4 months: Wt (*n* = 22), 5xFAD (*n* = 28); 8 Months: Wt (*n* = 22), 5xFAD (*n* = 25); 12 Months: Wt (*n* = 13), 5xFAD (*n* = 17) and 14 Months: Wt (*n* = 19), 5xFAD (*n* = 18). Correlation was assessed using Pearson’s coefficient. Inner plexiform layer (IPL), Inner nuclear layer (INL), Inner segments (IS), Outer plexiform layer (OPL), Outer nuclear layer (ONL), Outer segments (OS), Retinal ganglion cells (RGC), Retinal nerve fibre layer (RNFL), Retinal pigment epithelium (RPE)

OCT measurements were collected to evaluate any gross structural alterations in 5xFAD retinae (Fig. 3a). Volumetric cross-sectional data of individual retinal layers scanned over a 1.4mm^2^ area revealed no differences except a significantly thickened ONL (5xFAD: R^2^ = 0.3379, *p* = 0.304 and Wt: R^2^ = 0.6673, *p* = 0.091) and IS (5xFAD: R^2^ = 0.0601, *p* = 0.691 and Wt: R^2^ = 0.0576, *p* = 0.697) by 8 months relative to age-matched Wt littermates. However, with increasing ages at 10, 12 and 14 months, differences in the ONL and IS were no longer evident between the two cohorts (Fig. 3b). OCT measurements were also obtained by grouping retinal layers as inner (RNFL—INL), middle (OPL—ONL) and outer (IS—RPE). No differences were reported in the inner retinal layers between the groups. The thickness of the middle retinal layers that encompass the ONL, showed significant differences between the groups at 8 months which were abolished thereafter with age. Once values for IS-RPE layers (outer retina) were combined, the differences between the IS were no longer evident. The summed thicknesses of individual retinal layers (total retinal thickness) also showed no differences between Wt and 5xFAD groups. Irrespective of cohorts, all mice displayed a noticeable reduction in retinal thickness after 12 months, though these were not statistically significant.

### Longitudinal colour fundus photography and fluorescence angiography assessments of 5xFAD retinae followed by end-point histological analyses

**Fig. 4:**
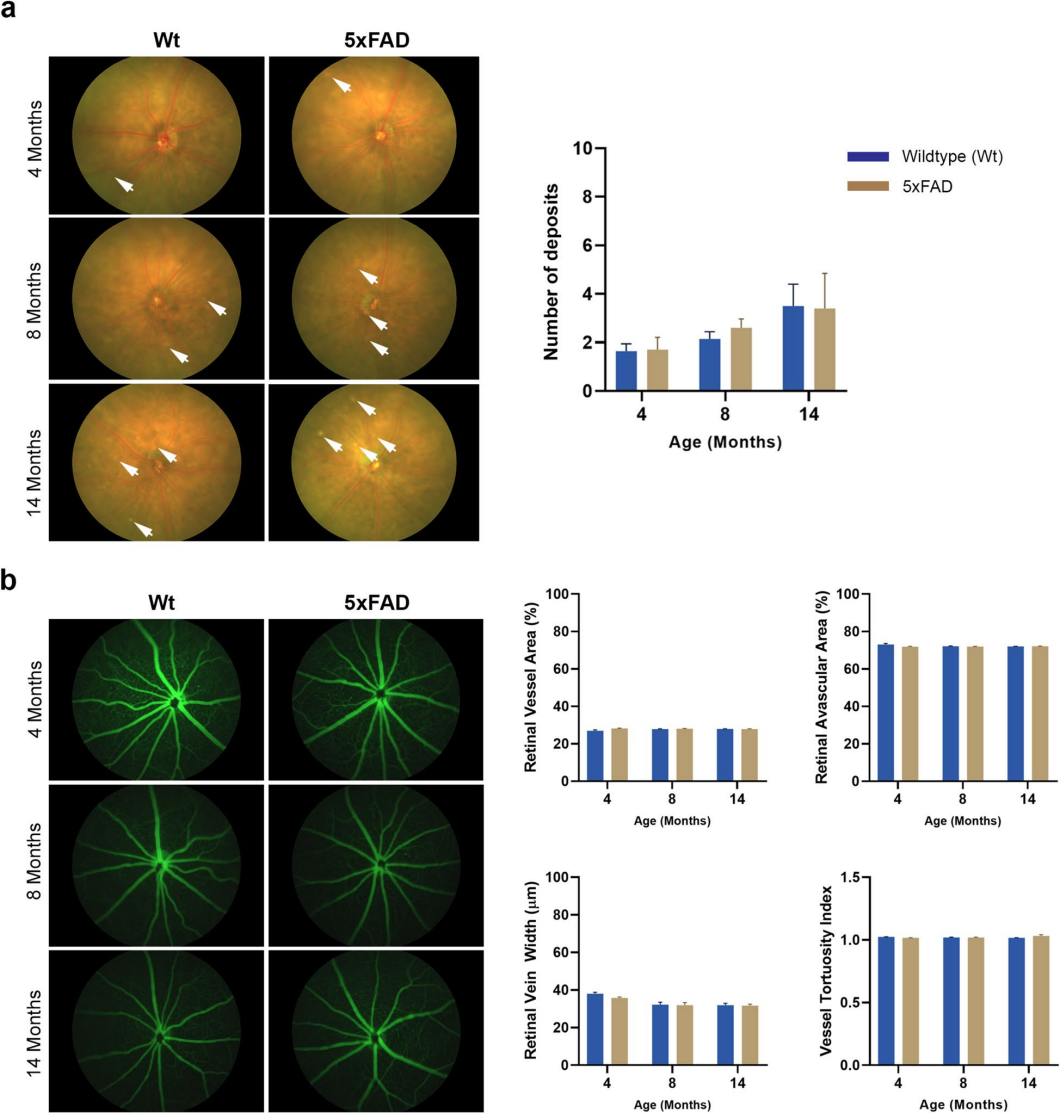
Funduscopy and fluorescein angiography images of 5xFAD and wildtype mouse retinae. **a** Anonymised colour fundus photography (CFP) taken at 4, 8 and 14 months were scrutinised for any evidence of pathology, which showed evidence of punctate/white subretinal deposits in both cohorts that gradually increased with age. **b** Fluorescein angiography (FFA) images were quantified in MATLAB using Quant BV software to assess retinal vessel area, the avascular region, vein width and vessel tortuosity. No differences were observed between 5xFAD and wildtype littermate mice. Similarly, longitudinal studies revealed no evidence of dye leakage, indicative of compromised retinal vessels in either group. Data is expressed as means ± SEM (standard error of the mean). Statistical comparisons were made using a two-way ANOVA with Bonferroni’s post-hoc test. Experimental replicates were as follows. 4 Months: Wt (*n* = 7), 5xFAD (*n* = 5); 4 months: Wt (*n* = 7) 5xFAD (*n* = 5); 8 months: Wt (*n* = 7), 5xFAD (*n* = 5) and 14 months: Wt (*n* = 5), 5xFAD (*n* = 5)

To detect any gross indicators of Aβ and/or age-related retinal pathology, retinae were imaged at 4, 8 and 14 months by CFP. We observed subretinal white dots in both 5xFAD and Wt littermates which gradually increased with age in both groups. There were no significant differences in the frequency of these deposits between the two groups (Fig. 4a). In order to determine whether any changes had occurred to retinal vessels, longitudinal FFA measurements were collected at the same timepoints. This allowed the quantification of retinal vessel area, width and tortuosity as well as the retinal avascular area. Assessments of anonymised images showed that there were no differences between groups, including any evidence of dye leakage from retinal vessels due to increased age and/or Aβ effects (Fig. 4b).

**Fig. 5:**
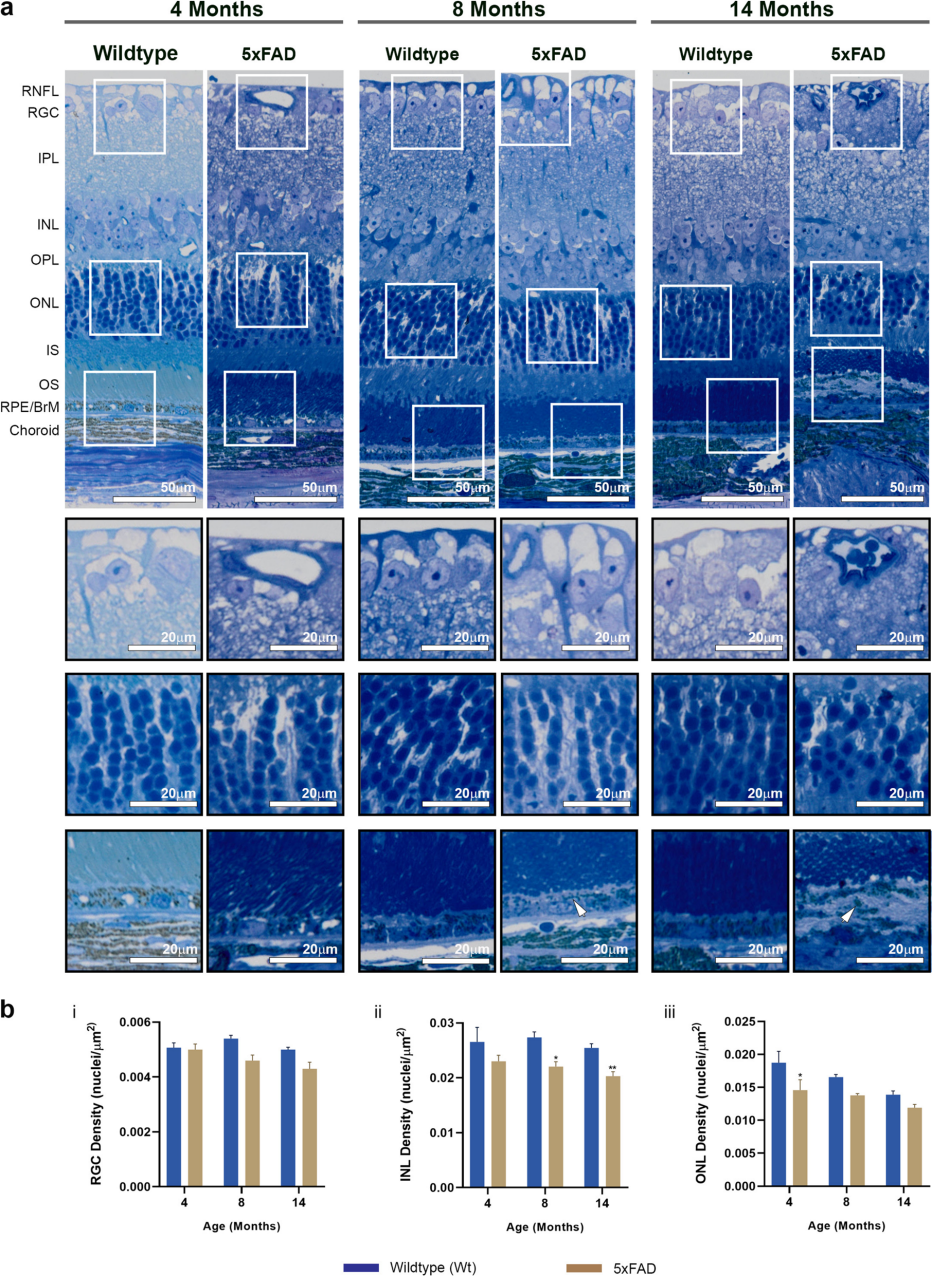
Light microscopy analyses of chorioretinal sections of 5xFAD and wildtype mice. **a** Representative images of wildtype mice and 5xFAD retinae at 4, 8 and 14 months, which were stained with toluidine blue showing cross-sections through the retina and associated tissues. Scale bars correspond to 50µm. Sections highlighted in white boxes are shown as magnified inserts which elicited closer scrutiny: RNFL—IPL (top panel), INL—ONL (middle panel) and IS—Choroid (lower panel). Occasionally, evidence of detached or multinuclear clusters were observed subretinally in 5xFAD eyes (white arrows). Scale bars in inserts correspond to 20µm. **b** Quantification of nuclei in the RGC layer in anonymised sections showed no significant differences between 5xFAD and wildtype retinae at any timepoint, though 5xFAD retinae contained fewer nuclei with increasing age. By contrast, analyses of the INL revealed significantly fewer bipolar cell nuclei in 5xFAD retinae compared to controls at 8 and 14 months. Scrutiny of the adjacent ONL revealed significantly fewer photoreceptor nuclei in 5xFAD retinae compared to wildtype mice at an earlier timepoint of 4 months. However, these differences became less evident afterwards, though 5xFAD mice contained fewer ONL nuclei compared to controls at 8 and 14 months. Data is expressed as means ± SEM (standard error of the mean). Statistical comparisons were made using a two-way ANOVA with Bonferroni’s post-hoc test with significance shown as * *p* ≤ 0.05 and ** *p* ≤ 0.01. Experimental replicates were as follows. 4 Months: Wt (*n* = 3), 5xFAD (*n* = 3); 8 Months Wt (*n* = 3), 5xFAD (*n* = 3) and 14 Months: Wt (*n* = 5), 5xFAD (*n* = 4). Bruch’s membrane (BrM), Inner plexiform layer (IPL), Inner nuclear layer (INL), Inner segments (IS), Outer plexiform layer (OPL), Outer nuclear layer (ONL), Outer segments (OS), Retinal ganglion cells (RGC), Retinal nerve fibre layer (RNFL), Retinal pigment epithelium (RPE)

Mice were killed at 4, 8 and 14–15 month intervals, and eyes enucleated for end-point histological analyses initially by light microscopy of toluidine blue stained retinal sections. Scrutiny of serially-sectioned chorioretinal tissues in anonymised mouse eyes showed no marked differences in the inner retinae of 5xFAD mice or Wt littermates. Occasionally, retinal vessels in the RNFL and RGC layer were visible in cross-section, some of which contained luminal red blood cells that were stained deep blue. Measurement of the RGC density (nuclei per µm^2^) showed no differences between 5xFAD and Wt cohorts at 4, 8 or 14 months (Fig. 5b i). By contrast, significantly diminished nuclear counts in the INL were recorded in 5xFAD mice compared to Wt littermates after 8 months (5xFAD: 0.022 ± 0.008, Wt: 0.026 ± 0.0006, *p* = 0.0093) (Fig. 5b ii). Interestingly, quantification of cell density in the ONL revealed reduced nuclei numbers in 5xFAD eyes at an earlier time point of 4 months compared to Wt littermates (5xFAD: 0.013 ± 0.0008, Wt: 0.164 ± 0.0014, *p* = 0.0283), which was abolished with increasing age (Fig. 5b iii), though we observed a trend of progressively thinning ONL in both groups over time. There were no other notable differences between the groups by light microscopy, except for occasional evidence of subretinal multinucleation and/or detached cells in 5xFAD eyes. However, these were challenging to identify in toluidine blue stained chorioretinal sections without further scrutiny.

Given that diminished nuclei in the INL were recorded in 5xFAD retinae compared to Wt littermate controls after 8 months, a proportion of chorioretinal sections were selected for further scrutiny by confocal microscopy. Tissues were labelled with anti-postsynaptic density 95 (PSD95), which identified postsynaptic terminals of bipolar cells in anonymised sections of mice aged 8 and 15 months. Mean grey values of PSD95 staining in anonymised chorioretinal sections of 8 month old mice were as follows: 5xFAD: 120.52 ± 9, Wt: 124.3 ± 16.4, *p* = 0.875 and at 15 months 5xFAD: 67.69 ± 0.05, Wt: 88.68 ± 5.61, *p* = 0.133 (raw data in Supplementary Table S4). Comparison of values amongst Wt littermates at 8 and 15 months revealed a diminishing pattern of PSD95 labelling (8 months Wt: 124.3 ± 16.4 vs. 15 months Wt: 88.68 ± 5.61, *p* = 0.067) which was mirrored in 5xFAD mice (8 months: 120.52 ± 9 vs. 15 months 67.69 ± 0.05, *p* = 0.333), but without any significant differences (Supplementary Figure S3).

### Ultrastructural scrutiny of 5xFAD chorioretinal tissues

**Fig. 6:**
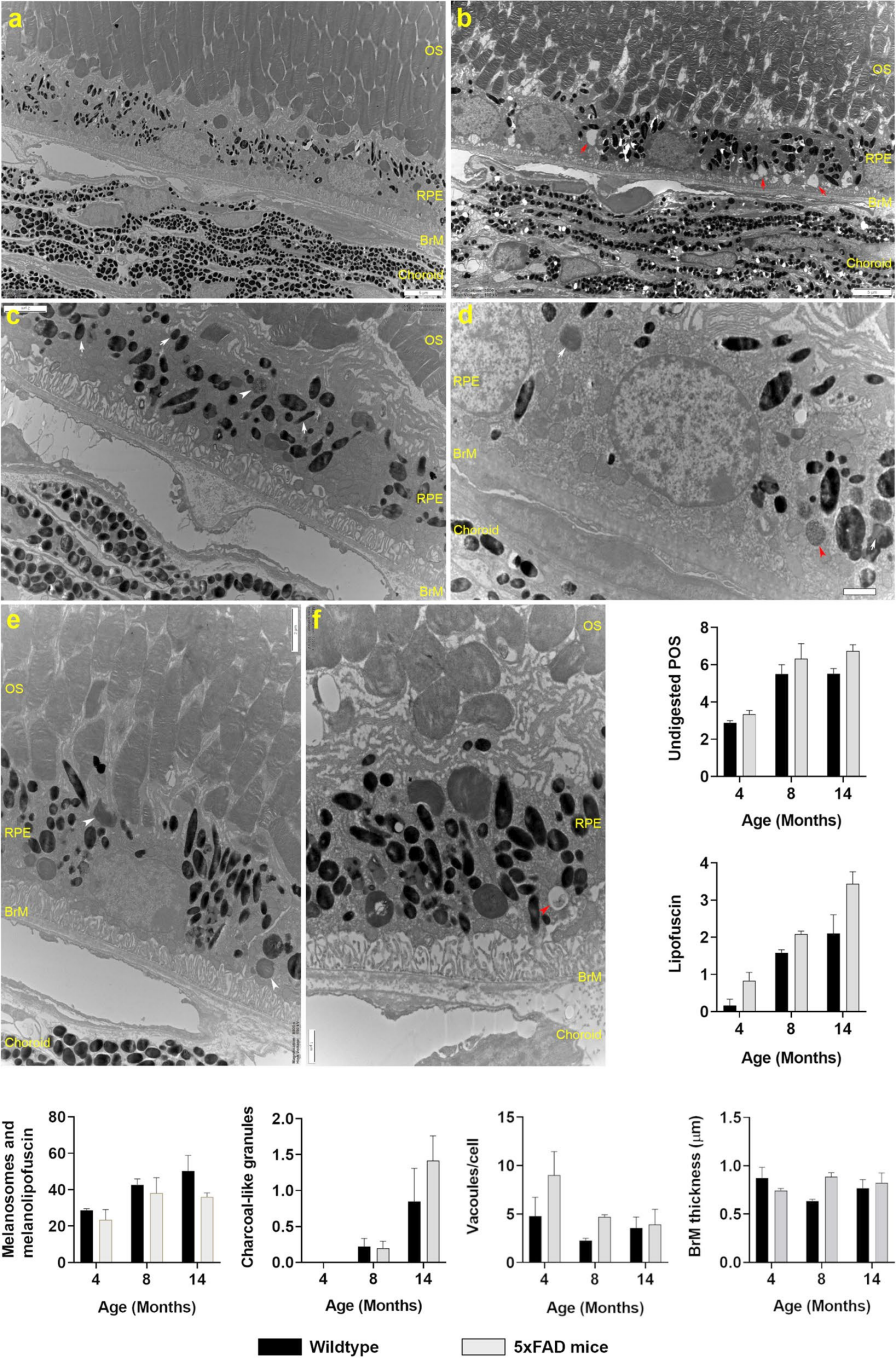
Ultrastructural comparison of 5xFAD and wildtype mouse retinae. Mice culled at 4, 8 and 14 months provided details of Aβ and/or age-related changes in each retinal layer. **a** A representative low-powered electron micrograph (EM) (× 3000) showing the cross-section of a 14 month old wildtype mouse retina. Wildtype littermates at this experimental end-point showed no evidence of any retinal abnormalities. **b** Scrutiny of the 5xFAD outer retina at 4 months as illustrated by this representative EM micrograph also showed no evidence of pathology, though intracellular vacuoles (red arrows) were prevalent. **c** EM micrograph of 8 month old 5xFAD outer retina, further magnified (× 8000) and focused on the RPE layer, showing undigested POS (white arrowhead) as well as electron-dense structures such as melanosomes (white arrows) in RPE cells. **d** Evidence of charcoal-like granules (red arrowhead) alongside electron-dense bodies including lipofuscin (white arrows) in RPE cells of a 14 month old 5xFAD eye. **e** Representative EM micrograph showing undigested POS (white arrowheads) amongst other electron-dense granules in 14 month old 5xFAD RPE cells. However, the apical RPE microvilli and their basolateral infolds appear normal in these transgenic mice. **f** High-powered representative EM micrograph (× 12,000) of 8 month old 5xFAD retina showing abundant electron-dense granules including intracellular vacuoles (red arrowhead) in RPE cells with no further evidence of any morphological abnormalities. Histograms showing the quantification of aforementioned features in anonymised sections revealed no significant differences between 5xFAD and wildtype littermates. However, the frequencies of most such features appear to increase with age in both groups. Quantification of BrM thicknesses revealed no differences between cohorts or across different age groups. Scale bars are indicated in each micrograph which corresponds to 5µm (a, b), 2µm (c, e) and 1µm (d, f). Bruch’s membrane (BrM); Outer segments (OS); Retinal pigment epithelium (RPE)

To assess Aβ and aged-related changes at the ultrastructural level, a proportion of enucleated eyes were scrutinised by conventional TEM. Micrographs were collected from serially-sectioned chorioretinal tissues that were cut parallel to the long axis of photoreceptors with the identity of their origins anonymised to eliminate bias in subsequent studies. Scrutiny of the inner retina revealed no marked differences between 5xFAD or Wt littermates over the study period. Our focus therefore shifted to studying the outer retina and adjacent choroidal tissues. TEM micrographs of Wt retinae at × 3000—× 12,000 magnification showed no pathology across their lifetime (Fig. 6a and Supplementary Figure S4). Scrutiny of the 5xFAD outer retina at 4 months also showed no evidence of pathology, though high levels of intracellular vacuoles within RPE cells were noticeable even at this early stage (Fig. 6b). No morphological differences in the ONL, IS or OS were evident between the 2 groups. Similarly, the RPE layer, including its apical microvilli and basolateral infolds, were comparable between 5xFAD and Wt littermates. However, undigested photoreceptor outer segments (POS) which appear as fingerprint-like contours within membranes, lipofuscin, melanosomes and melanolipofuscin were observed at higher frequencies in the RPE of 8 and 14 month old 5xFAD retinae. We also noted a higher incidence of charcoal-like granules, which had been reported by others in older animals, particularly in 5xFAD eyes (Fig. 6c-f and Supplementary Figure S4). RPE cells of 5xFAD mice also contained a greater number of vacuoles compared to their Wt counterparts. Quantification of these features in anonymised chorioretinal sections revealed a pattern of increasing frequencies correlated with age, broadly with more prevalence in 5xFAD RPE compared to Wt controls but with no significant differences (Fig. 6). Bruch’s membrane (BrM) thickness measurements revealed no differences between groups at 4 months (5xFAD: 0.744 ± 0.036 SD and Wt: 0.874 ± 0.19) and 8 months (5xFAD: 0.89 ± 0.07 SD and Wt: 0.636 ± 0.03) or at 14 months (5xFAD: 0.824 ± 0.2 SD and Wt: 0.768 ± 0.2) (Fig. 6). Scrutiny of choroidal cross-sections also showed no morphological differences between the two groups at any timepoint.

## Discussion

Recent advances in the treatment of AD with Aβ-targeting approaches have ushered a renewed interest in the correlation between Aβ and neurodegeneration [30–32]. Although studies to date suggest that only a proportion of AD patients could benefit, these developments nonetheless offer new hope to those where no effective treatments have been developed for over 20 years. The pathogenic effects of Aβ however are not only limited to the neuropathology of the brain [7, 33], but play a role in the development of retinopathies such as AMD, which also have no effective treatments. Histopathological studies in chorioretinal tissues of aged and AMD donor eyes show the accumulation of Aβ, particularly localised in subretinal drusen and correlated with advanced stages of retinopathy. Retinal Aβ accumulation is also reproduced in numerous transgenic rodent models that were primarily developed to investigate AD. Studies in these models have revealed the identity of specific retinal cells/layers that synthesise Aβ, showing patterns of Aβ production and deposition in ocular tissues [34]. To understand Aβ effects in the living retina, our previous work used subretinally injected nanomolar concentrations of human oligomeric Aβ_1–42_ in C57BL6/J Wt mouse eyes to produce salient AMD-like features that developed rapidly within 2 weeks [20, 35, 36]. This approach revealed the extent of damage caused to retinal tissues by a physiologically-relevant dose of a single Aβ species. Although acute models of this kind circumvent the need to age rodents for long periods before developing a deleterious retinal phenotype, transgenic mice offer the possibility of studying chronic age-related retinal Aβ toxicity in a more physiologically-relevant manner. Transgenic mice have also been used to demonstrate the importance of Aβ in retinal diseases by impairing or preventing retinal Aβ activity, which resulted in a rescue of a deleterious retinal phenotype [37, 38]. Indeed, compared to acute models, transgenic mice may better recapitulate the chronic Aβ exposure as well as its complex behaviour, where different Aβ species exist in interchangeable aggregation states, each with specific toxic effects [39]. Compared to other transgenic AD models, 5xFAD mice express high levels of ocular Aβ, which makes these an attractive model for such studies [19, 40]. Although others had previous reported Aβ toxicity in the 5xFAD retina, we wanted to exclude any confounding effects exerted by genes linked with retinal degeneration that are often carried in these mice; an issue that investigators have only started to consider. For instance, several recent studies involving 5xFAD mice excluded presence of the *Pde6b*^*RD1*^ allele [34, 40–45]. We performed a further screening of retinal degeneration genes *Pde6b*^*RD8*^, *Agouti*, *Tyr* and *Oca2*, including *Pde6b*^*RD1*^ to exclude any confounding effects which could be caused by their presence [28, 29]. The *rd* gene encodes a transcript for the β-subunit of the phosphodiesterase enzyme complex that participates in the cGMP-mediated phototransduction cascade. Mutations in this gene are reported in autosomal recessive retinitis pigmentosa patients which bears a phenotypic resemblance to pathology caused by the mouse *Pde6b*^*RD1*^ mutation. As *rd* mutations are common in mice, it is important to avoid murine strains or stocks carrying the *rd1* allele in retinal studies [46, 47]. Mice homozygous for *rd8* also show retinal degeneration but in addition develop large white retinal deposits as well as reduced A and B waves that remain stable for 1 year before showing a further loss in amplitude [47]. Humans lacking a functional *p* protein have oculocutaneous albinism type 2 (OCA2). Mice lacking this protein develop pink eyes and a light grey fur coat. Melanocytes from *p*-deficient mice or OCA2 patients contain small, minimally pigmented melanosomes [48]. *Tyr* and *Oca2* are responsible for the two most common forms of OCA (OCA1 and OCA2, respectively). Mice with mutations in either of these genes are commonly used as models of OCA. For example, *Tyr_c2j* mice have diminished RGC, IPL and RPE thicknesses, reduced photoreceptor lengths by 16 weeks, as well as abnormally high photoreceptor death [49, 50]. *Oca2* is also carried in strains such as SJL/J [51]; the (C57BL/6 × SJL) F1 strain of origin for 5xFAD mice (MMRRC Strain #034848-JAX) and is therefore another important retinal degeneration gene to avoid in studies of other retinal disorders. By excluding these deleterious genes, our findings were able to identify bona fide features of retinopathy solely mediated by Aβ from any confounding effects and from wider age-related retinal changes.

Regular assessment of living retinas by ERG, optometry, OCT, CFP and FFA also provided 3Rs advantages, enabling data gathering using smaller cohorts over longer periods, which significantly reduced animal numbers that would have been used otherwise. Of these, ERG measurements showed a general decline of retinal activity in both cohorts with increasing age. However, A and B waves were significantly diminished in 5xFAD mice at 8 months compared to their Wt littermates, though the general age-related decline in ERG responses in all animals appeared to abolish these differences subsequently. Another study also reported diminished B waves at 6 months even under lower luminescence stimuli [38], though others showed no functional ERG deficits in this model [41, 52]. It appears that differences in the luminescence levels of the stimuli, whether measurements were taken under dark or light adapted conditions, as well as age, contribute to contrasting ERG results [42, 43]. Our optokinetic tracking experiments, which is a measure of visual acuity and function, showed a gradual decline with advancing age in all mice. However, we recorded a significant reduction in the ability to track clockwise, counter-clockwise, up or down movements in 14 month old 5xFAD mice compared to age-matched controls. Another study also reported optokinetic deficiencies in 5xFAD retinae but at an earlier timepoint of 6 months [41]. Scrutiny of retinae by OCT in our study revealed no marked differences between cohorts except for thickened ONL and IS layers in 5xFAD eyes at 8 months. The thickness of the ONL in 5xFAD retinae appear to be substantially greater than in Wt counterparts, sufficient to translate to significant differences even when OPL—ONL layers were grouped together. Others reported a pattern of RNFL thinning in 5xFAD eyes, which outweighed IPL thickening that manifested as an overall thinning of the RGC [42]. Another study that scrutinised DAPI-stained chorioretinal sections, reported a thicker ONL in 4 month old 5xFAD mice with differences emerging in the total retinal thickness which increased at 6 months [41]. Interestingly, the use of a custom designed spectral domain OCT revealed retinal thinning in 6 month old 5xFAD mice with regional differences in the dorsal and temporal quadrants [44]. Collectively, these findings indicate that in some instances conventional OCT may be insufficiently sensitive to detect subtle and/or regional structural differences in 5xFAD retinae associated with ageing or pathology as may be anticipated. Indeed, a location-specific OCT analysis of the human retina showed that RNFL thickness displayed an outwards radial pattern from the nasal retina. By contrast, all other layers from the GCL to the RPE displayed concentric patterns of thickness decreasing from the fovea. Similar rates of age-related thickness change were observed in the ganglion cell layer, IPL, INL, and ONL, while the IS/OS demonstrated lesser rates of thickness decline [53]. Nonetheless, for mouse studies of this kind, excluding retinal generation genes remains an important caveat.

The mouse fundus assessed by CFP in our studies, showed no differences between 5xFAD and Wt littermates, except for a gradual increase in subretinal deposits in the form of well-defined yellowish/white dots that increased with age. Such deposits have also been reported in other mouse models displaying features of retinal degeneration [54, 55]. However, these were infrequently observed in our study, compared to those with a similar appearance that were abundantly observed in the Cxcr5 chemokine receptor knockout mice [56]. Subretinal deposits in Cxcr5^−/−^ mice also became more frequent with age where some fused with each other and were also detected at the light microscopy (LM) level and by TEM. Though superficially drusen-like in appearance, these deposits may be molecularly distinct from drusen due to important anatomical and physiological differences between rodents and primates. Though we found no evidence of these deposits in toluidine blue stained tissues or at ultrastructural level, perhaps due their infrequency, advanced age rather than retinal Aβ appears to be the primary factor in their occurrence. Evaluation of retinal vessel structure/morphology by FFA also showed no differences between cohorts in vessel area, width, vessel tortuosity or the retinal avascular area. Moreover, we found no evidence of compromised vessel integrity (dye leakage) in adult mice. However, recent findings in 5xFAD mice showed increasing Aβ accumulation in retinal vessels associated with the loss of pericytes with age [57]. We were therefore surprised that these observations did not appear to translate to any widespread morphological vessel parameters quantified in our study. However, the use of higher-resolution scanning techniques may have the potential to reveal alterations to the retinal vasculature of 5xFAD mice [40, 45], though findings from studies primarily focused on brain pathology in these mice, where the exclusion of retinal degeneration genes are not required, cannot necessarily be applied to assessments of their retinae.

Mice killed at selected timepoints along their lifecourse enabled histological studies to obtain further insights into these events. The first end-point at 4 months allowed scrutiny of the fully developed young/adult mouse retina [58]. The second end-point at 8 months (intermediary timepoint) provided a snapshot of the older retina, whilst the final 15 month end-point extended the scope of our longitudinal investigation to include the aged retina. To our knowledge, there are only a handful of other studies that examined 5xFAD mice older than 15 months. The first description of the 5xFAD model investigated animals until 16 months of age, but exclusively in the brain [18]. A more recent report phenotypically characterised its brain pathology across the lifespan until 18 months [59]. The oldest 5xFAD mice, where retinal pathology was studied, were 17 months of age [42]. In our experience, the susceptibility of older mice to repeated anaesthesia coupled with a severe AD phenotype, makes studies of aged 5xFAD challenging. Such animal welfare considerations necessitate establishing large cohorts at the outset, particularly if multiple end-points along their lifecourse combined with different experimental pipelines such as those in our study, requires subsequent splitting of cohorts into smaller sub-groups. Scrutiny of toluidine blue stained chorioretinal sections revealed significantly diminished nuclei numbers of bipolar cells in 5xFAD mice after 8 months. We also observed significantly fewer photoreceptor nuclei in 4 month old 5xFAD eyes, through subsequent reductions to nuclei counts of photoreceptors in both cohorts abolished these differences as mice aged. Previous studies have reported the accumulation of Aβ in the retinae of 5xFAD mice [60–62], including retinorecipient areas which showed synaptic loss and neuronal degeneration [34]. Though our studies did not show further evidence of Aβ-mediated retinopathy at confocal resolution, diminishing PSD95 labelling in the INL suggested impairment of photoreceptor-bipolar connectivity as mice aged, consistent with reduced retinal thicknesses in both cohorts by OCT, particularly after 12 months. Abnormalities in retinal neurons were also consistent with impaired microtubule associated protein 2 (MAP-2) labelling which we reported earlier in precursor retinal neurons following acute Aβ exposure [63]. Scrutiny of the inner retina at ultrastructural resolution however did not reveal further differences between 5xFAD and Wt eyes. As changes in the outer retina and choroid are frequently associated with retinal degeneration, we also examined these tissues by TEM. We found no obvious morphological abnormalities in photoreceptor, RPE, BrM or choroidal layers of 5xFAD mice vs. their Wt littermates. The apical RPE microvilli and RPE basolateral infolds in 5xFAD eyes appeared to be normal across the lifecourse though others had reported abnormalities in these structures ultrastructurally [64]. Scrutiny of electron-dense granules, as described within the RPE in histopathological studies of donor human eyes consisting of lipofuscin, melanolipofuscin and melanosomes [65], also showed no differences between cohorts. However, these granules, alongside the presence of undigested POS which can be discerned as membrane-bound fingerprint-like intracellular structures, appeared to increase in frequency with advanced age. We also observed the increased presence of charcoal-like granules within RPE, which had been described in 5xFAD mice [64], associated with advanced age in both cohorts of our study. RPE vacuoles that indicate pathology were reported in 5xFAD eyes at LM resolution by others [61]. Although we also observed RPE vacuoles at 4 months by TEM, particularly in 5xFAD retinae, they became less evident as 5xFAD and Wt mice aged. Similarly, BrM abnormalities in 5XFAD eyes were reported by others [64], though TEM analysis showed no evidence of BrM abnormalities in our study. We were therefore initially surprised that chronic Aβ exposure did not lead to more widespread pathology in the outer retina, particularly as we and others had shown toxic Aβ effects in cultured RPE [20, 66–68]. However, subtler and sometimes transient Aβ effects in the 5xFAD retina, which we report in our study, were consistent with the chronic Aβ-burden in aged/AMD eyes with the caveat that Aβ-rich drusen present in aged/AMD retina is an important missing element in this mouse model. Indeed, our recent work, where a US Food and Drug Administration-approved experimental drug for AD was re-purposed, demonstrated a rescue effect in RPE cells from Aβ-mediated toxicity [69, 70] that led to the Phase 2 MAGNIFY trial (ClinicalTrials.gov ID: NCT05893537) for the treatment of GA AMD.

## Conclusions

**Fig. 7:**
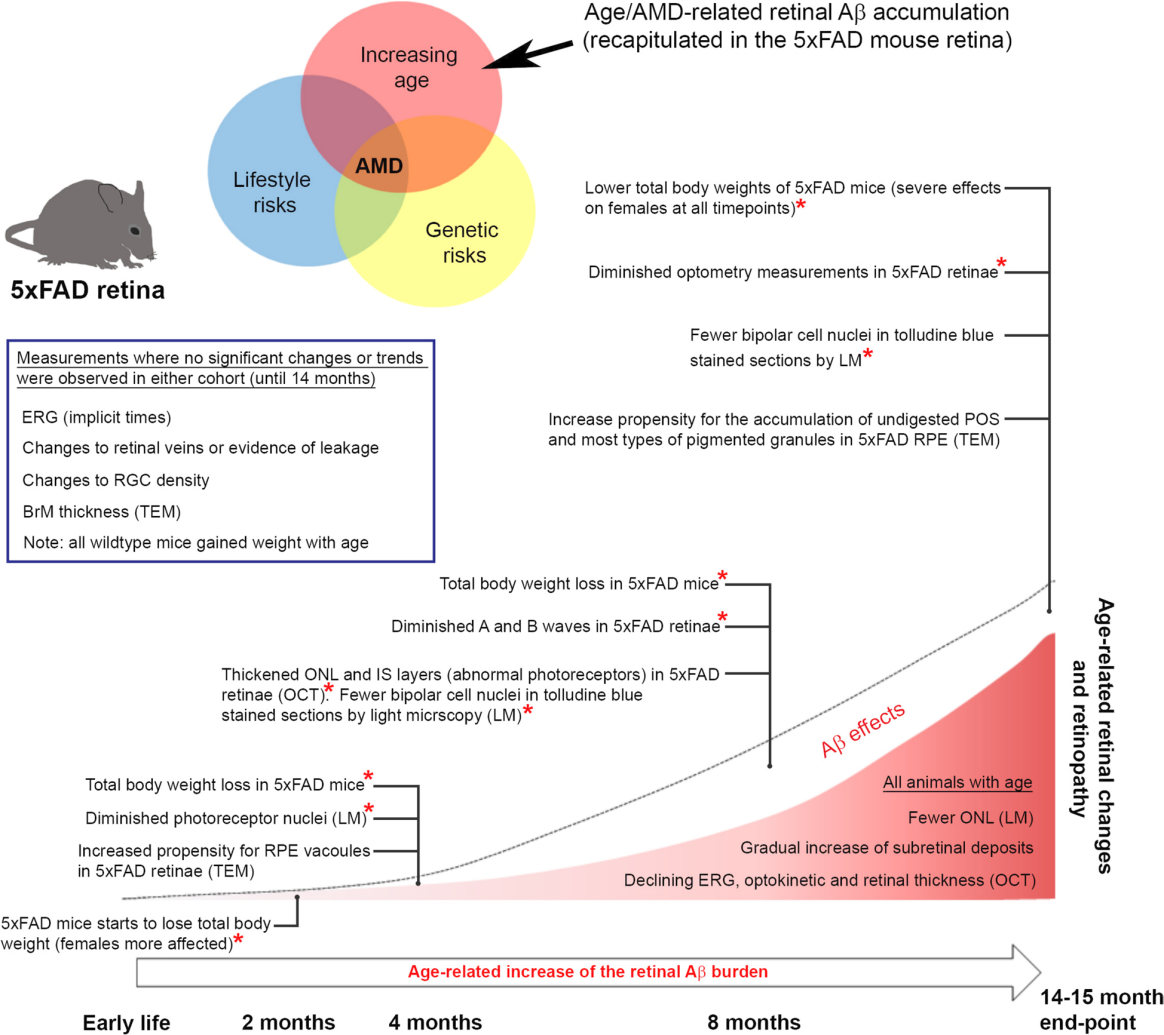
Study concept and summary of changes in 5xFAD mice retinae driven by Amyloid beta (Aβ) pathology and age. Venn diagram: Age-related macular degeneration (AMD) is caused by the confluence of advanced age combined with genetic as well as lifestyle risk factors. Although AMD is not associated with any known mutations in the amyloid precursor protein (*APP*) or presenilin-1 (*PSEN1*) genes which are over-expressed in 5xFAD mice, the resulting high levels of retinal Aβ effectively recapitulates the age/AMD-associated Aβ-burden in the human retina. Schematic of summary findings: Lifecourse of 5xFAD mice showing key experimental timepoints correlated with notable features of Aβ-driven retinopathy over any age-related changes. Statistically significant differences are indicated by a red asterisk. Age-related changes are also noted in both cohorts. Measurements which produced no obvious changes/trends in either cohort are listed within the box. Light microscopy (LM) and transmission electron microscopy (TEM), indicate where data were obtained using these approaches. Amyloid beta (Aβ); Bruch’s membrane (BrM); electroretinography (ERG); inner segments (IS); optical coherence tomography (OCT); outer nuclear layer (ONL); photoreceptor outer segments (POS) and retinal pigment epithelium (RPE)

In summary, our study delineated Aβ-mediated retinal pathology from wider changes in the retina due to effects of ageing. These findings were strengthened by the elimination of potentially confounding influences caused by retinal degeneration alleles in 5xFAD mice. Our results show an evolving and complex picture, where in a backdrop of age-related changes, the growing retinal Aβ-burden led to the development of important retinal degeneration features (Fig. 7). Age-related alterations in the 5xFAD retina were also consistent with those reported as the retina ages, including diminished retinal function, alterations to its structure, loss of synaptic connectivity and the accumulation of electron-dense RPE inclusions [71]. Our study also highlighted advantages and limitations of this model, including early weight loss which adversely affected female 5xFAD mice in particular. Though this did not necessarily translate to the loss of animals, even non-invasive procedures involving repeated anaesthesia had to be limited in older mice with a severe AD-like phenotype. Chronic Aβ exposure led to the development of retinopathy including diminished photoreceptor nuclei (4 months) alongside reduced A and B waves at 8 months, which however, became abolished with age. From 8 months onwards, Aβ exposure also resulted in fewer bipolar cells and diminished retinal functionality (OKT) by 14 months. These occurred with advancing age, where features including increasing subretinal deposits and elevated electron-dense granules as well as undigested POS within RPE were recorded in all mice. Ageing was also correlated with fewer ONL as well as declining ERGs, optokinetic and retinal thickness measurements in both cohorts. By contrast, no differences between groups were recorded in RGC density or in ERG implicit times. Retinal vessels also showed no marked changes. No pathology was detected in the 5xFAD BrM, consistent with our previous observations that even acute Aβ exposure by subretinal injection failed to elicit a neovascular phenotype in wildtype mice [20]. To our knowledge, the findings reported here are obtained from the largest group of 5xFAD mice describing Aβ-mediated retinal pathology to date. Chronic Aβ exposure in the 5xFAD retina does not appear to result in wholescale pathology, but rather lead to the earlier development of specific retinal degeneration characteristics, consistent with evidence from AMD patients showing that retinal Aβ is only one contributing factor to disease. The identification of these features advances our understanding of how Aβ contributes to multifaceted retinopathies.

## Supplementary Information

Supplementary Material 1.

Supplementary Material 2.

## Data Availability

Availability of data and materials All data have been included in the article and in the supplementary material. Reasonable requests for raw data will be considered by the authors before being made available to third parties.

## Abbreviations

Aβ: Amyloid beta
AD: Alzheimer’s disease
AMD: Age-related macular degeneration
BrM: Bruch’s membrane
CFP: Colour fundus photography
ERG: Electroretinography
FFA: Fundus fluorescein angiography
GA: Geographic atrophy
IPL: Inner plexiform layer
INL: Inner nuclear layer
IP: Intraperitoneal (injection)
IS: Inner segments
LM: Light microscopy
MCI: Mild cognitive impairment
OCT: Optical coherence tomography
OKT: Optokinetic tracking
OPL: Outer plexiform layer
ONL: Outer nuclear layer
OS: Outer segments
PSD95: Postsynaptic density 95
RGC: Retinal ganglion cells
RNFL: Retinal nerve fibre layer
ROI: Region of interest
RPE: Retinal pigment epithelium
TEM: Transmission electron microscopy
VEGF: Vascular endothelial growth factor
3Rs: Reduce, replace and refine the use of animals in research
5xFAD: Familial Alzheimer’s disease
