# Supplementary material for: A longitudinal study of the 5xFAD mouse retina delineates Amyloid beta (Aβ)-mediated retinal pathology from age-related changes
Source: Alzheimers Res Ther. 2025 Jun 19;17:136. doi: 10.1186/s13195-025-01784-w (PMC12177965; doi:10.1186/s13195-025-01784-w)
Supplement: Supplementary file 1 — Supplementary Material 1. [file 13195_2025_1784_MOESM1_ESM.pdf]

## Supplementary Figures

a

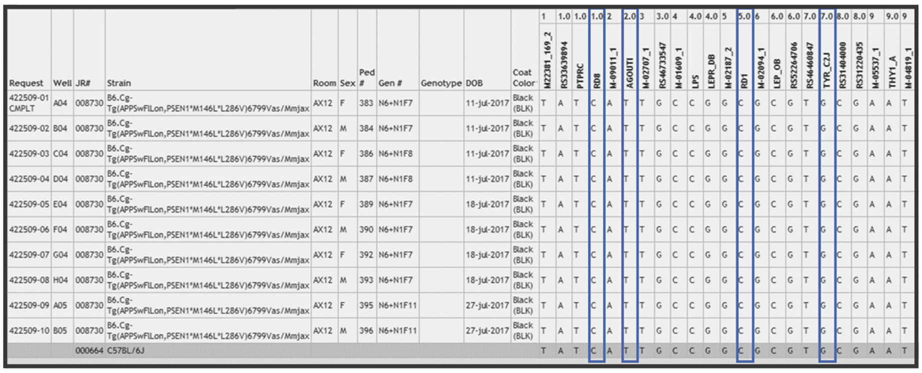

| | | | | | | | | | | | 1 | 1.0 | 1.0 | 1.0 | 2 | 2.0 | 3 | 3.0 | 4 | 4.0 | 4.0 | 5 | 5.0 | 6 | 6.0 | 6.0 | 7.0 | 7.0 | 8.0 | 8.0 | 9 | 9.0 | 9 |
|---|---|---|---|---|---|---|---|---|---|---|---|---|---|---|---|---|---|---|---|---|---|---|---|---|---|---|---|---|---|---|---|---|---|
| Request | Well | JR# | Strain | Room | Sex | Ped # | Gen # | Genotype | DOB | Coat Color | M22381_169_2 | RS33639894 | PTPRC | RD8 | M-09011_1 | AGOUTI | M-02707_1 | RS46733547 | M-01609_1 | LPS | LEPR_DB | M-02187_2 | RD1 | M-02094_1 | LEP_OB | RS52264706 | RS46460847 | TYR_C2J | RS31404000 | RS31220435 | M-05537_1 | THY1_A | M-04819_1 |
| 422509-01 CMPLT | A04 | 008730 | B6.Cg-Tg(APPSwFlLon,PSEN1*M146L*L286V)6799Vas/Mmjax | AX12 | F | 383 | N6+N1F7 | | 11-jul-2017 | Black (BLK) | T | A | T | C | A | T | T | G | C | C | G | G | C | G | C | G | T | G | C | G | A | A | T |
| 422509-02 | B04 | 008730 | B6.Cg-Tg(APPSwFlLon,PSEN1*M146L*L286V)6799Vas/Mmjax | AX12 | M | 384 | N6+N1F7 | | 11-jul-2017 | Black (BLK) | T | A | T | C | A | T | T | G | C | C | G | G | C | G | C | G | T | G | C | G | A | A | T |
| 422509-03 | C04 | 008730 | B6.Cg-Tg(APPSwFlLon,PSEN1*M146L*L286V)6799Vas/Mmjax | AX12 | F | 386 | N6+N1F8 | | 11-jul-2017 | Black (BLK) | T | A | T | C | A | T | T | G | C | C | G | G | C | G | C | G | T | G | C | G | A | A | T |
| 422509-04 | D04 | 008730 | B6.Cg-Tg(APPSwFlLon,PSEN1*M146L*L286V)6799Vas/Mmjax | AX12 | M | 387 | N6+N1F8 | | 11-jul-2017 | Black (BLK) | T | A | T | C | A | T | T | G | C | C | G | G | C | G | C | G | T | G | C | G | A | A | T |
| 422509-05 | E04 | 008730 | B6.Cg-Tg(APPSwFlLon,PSEN1*M146L*L286V)6799Vas/Mmjax | AX12 | F | 389 | N6+N1F7 | | 18-jul-2017 | Black (BLK) | T | A | T | C | A | T | T | G | C | C | G | G | C | G | C | G | T | G | C | G | A | A | T |
| 422509-06 | F04 | 008730 | B6.Cg-Tg(APPSwFlLon,PSEN1*M146L*L286V)6799Vas/Mmjax | AX12 | M | 390 | N6+N1F7 | | 18-jul-2017 | Black (BLK) | T | A | T | C | A | T | T | G | C | C | G | G | C | G | C | G | T | G | C | G | A | A | T |
| 422509-07 | G04 | 008730 | B6.Cg-Tg(APPSwFlLon,PSEN1*M146L*L286V)6799Vas/Mmjax | AX12 | F | 392 | N6+N1F7 | | 18-jul-2017 | Black (BLK) | T | A | T | C | A | T | T | G | C | C | G | G | C | G | C | G | T | G | C | G | A | A | T |
| 422509-08 | H04 | 008730 | B6.Cg-Tg(APPSwFlLon,PSEN1*M146L*L286V)6799Vas/Mmjax | AX12 | M | 393 | N6+N1F7 | | 18-jul-2017 | Black (BLK) | T | A | T | C | A | T | T | G | C | C | G | G | C | G | C | G | T | G | C | G | A | A | T |
| 422509-09 | A05 | 008730 | B6.Cg-Tg(APPSwFlLon,PSEN1*M146L*L286V)6799Vas/Mmjax | AX12 | F | 395 | N6+N1F11 | | 27-jul-2017 | Black (BLK) | T | A | T | C | A | T | T | G | C | C | G | G | C | G | C | G | T | G | C | G | A | A | T |
| 422509-10 | B05 | 008730 | B6.Cg-Tg(APPSwFlLon,PSEN1*M146L*L286V)6799Vas/Mmjax | AX12 | M | 396 | N6+N1F11 | | 27-jul-2017 | Black (BLK) | T | A | T | C | A | T | T | G | C | C | G | G | C | G | C | G | T | G | C | G | A | A | T |
| | | 000664 | C57BL/6J | | | | | | | | T | A | T | C | A | T | T | G | C | C | G | G | C | G | C | G | T | G | C | G | A | A | T |

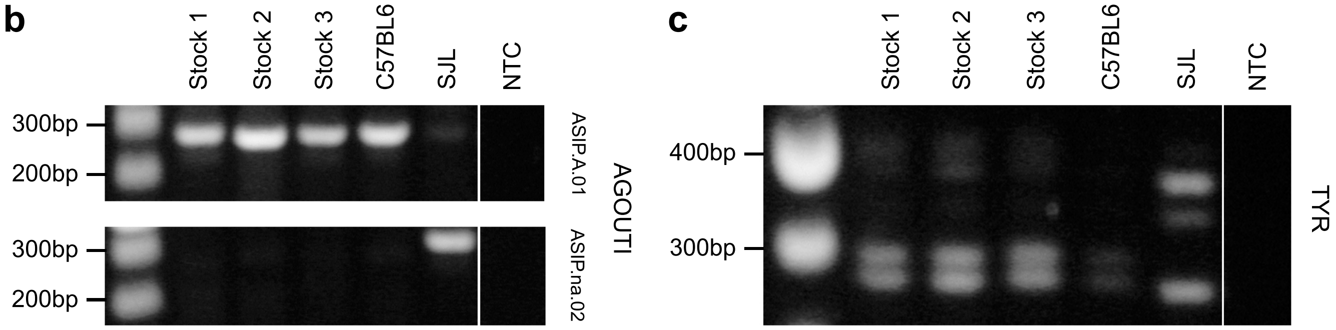


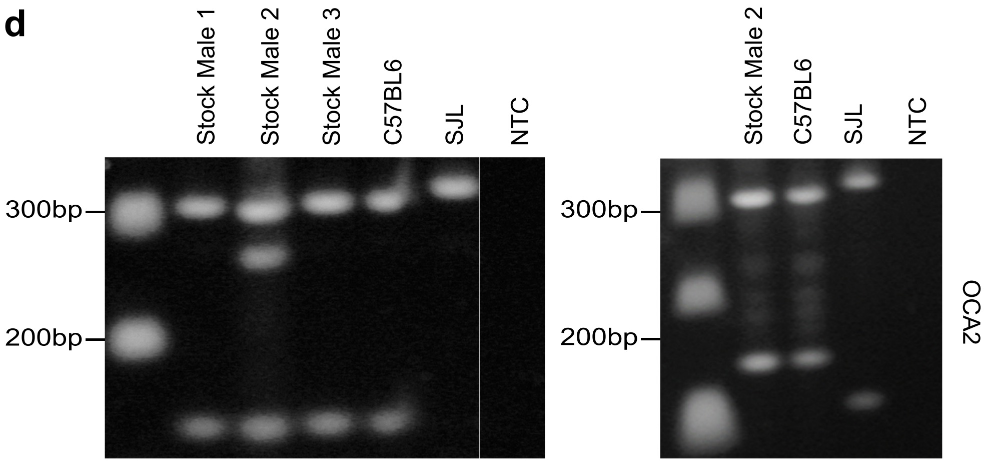


e

| Stock Male | Agouti | Oca2 | Tyr |
|---|---|---|---|
| 1 | a/a | Wild-Type | Wild-Type |
| 2 | a/a | Wild-Type | Wild-Type |
| 3 | a/a | Wild-Type | Wild-Type |

**Supplementary Figure S1: Screening of 5xFAD [B6.Cg-Tg(APPSwFlLon,PSEN1*M146L*L286V) 6799Vas/Mmjax] stock males for retinal degeneration alleles. (a)** Data obtained from Jackson laboratories showing a comparison of *Pde6b*$^{RD1}$, *Pde6b*$^{RD8}$, *Agouti* and *Tyr*_c2j in 5xFAD mice relative to a C57BL6/J reference sample genome. *Oca2* was not tested but was predicted to co-segregate on chromosome 7 due to close physical proximity. Upon receipt, stock males were subject to in-house PCR and restriction digest analyses to confirm the status of *Agouti*, *Oca2* and *Tyr* genotypes prior to commencing breeding of the colony. **(b)** PCR results determining the *Agouti* genotype. A band of 290bp is anticipated for wildtype mice in the case of ASIP.A.01 and 280bp for ASIP.na.02 if an Agouti allele is present. **(c)** Result of restriction digests determining the *Tyr* genotype. Bands of 393bp, 370bp and 78bp are anticipated in mice that are wildtype for *Tyr*. **(d)** Results of restriction digests for *Oca2*. Bands of 310bp and 146bp are anticipated in mice that are wildtype for *Oca2*. **(e)** Summary of PCR data confirmed that all three stock males purchased from Jackson Laboratories for generating the 5xFAD mouse colony were devoid of any mutations in the *Agouti*, *Oca2* and *Tyr* genes.

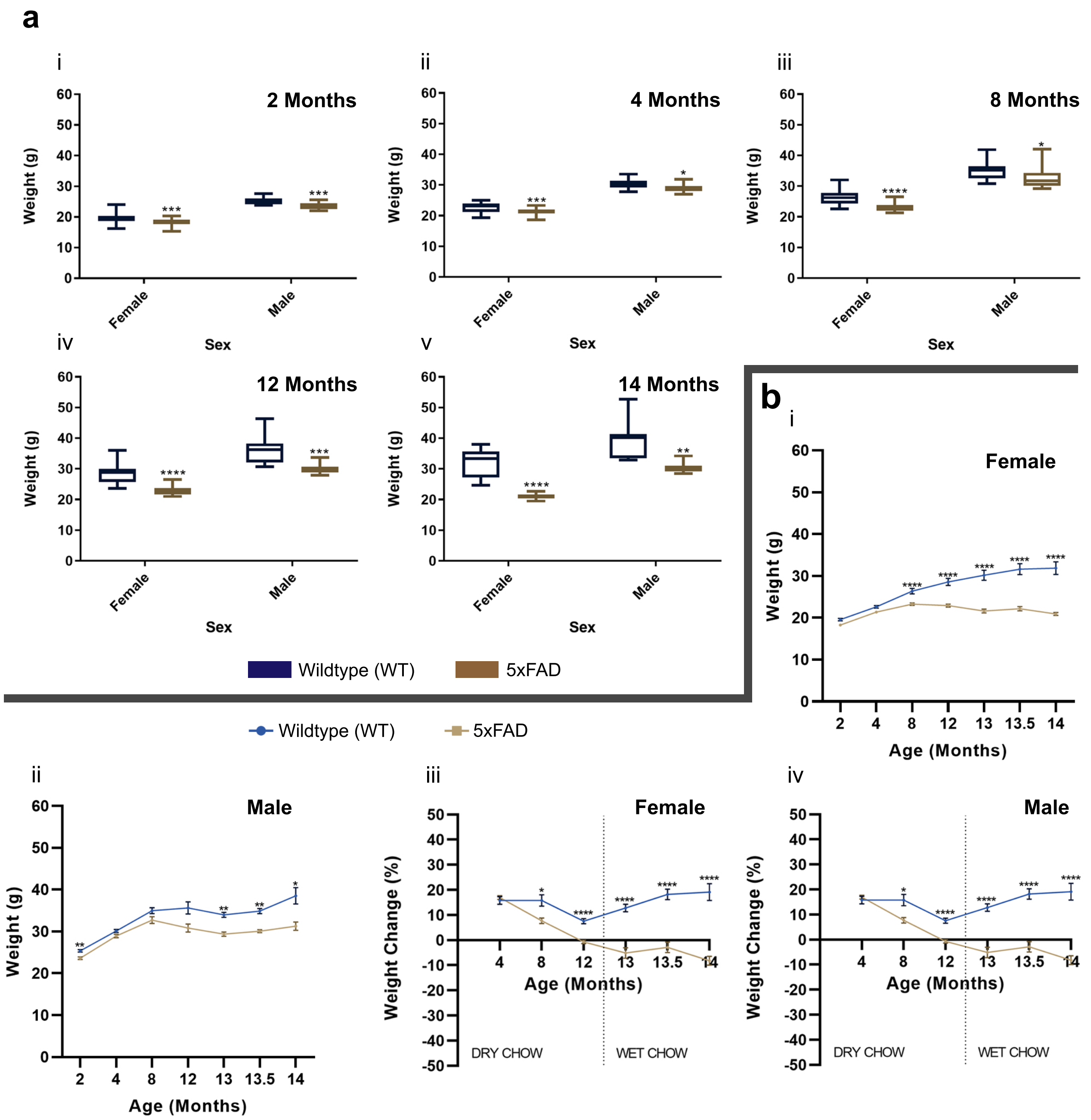


**Supplementary Figure S2: Longitudinal weight measurements of 5xFAD and wildtype littermates.** Transgenic 5xFAD mice weighed significantly less than control animals at all time points, with differences becoming more apparent after the age of 8 months. **(a)** Box and whisker plots showing average body weight where the error bars indicate maximum and minimum values with box-boundaries denoting the upper and lower quartiles. **(b)** Graphs showing the average and percentage change in body weight in 5xFAD mice and wildtype littermates over time. Weight change was calculated relative to the previous time point up to 8 months of age, after which this was calculated relative to the 8 month measurement when mice were deemed to have reached their mature/adult weight. Soft chow was offered *ad libitum* in addition to dry chow after 12.5 months to improve animal welfare and negate any significant reduction in body weight. Data is plotted as mean ± SEM. Statistical comparisons were made using a two-tailed unpaired t-test (a) and two-way ANOVA with Sidak's multiple comparisons (b), where statistical significance is indicated as * $p \leq 0.05$, ** $p \leq 0.01$, *** $p \leq 0.001$, **** $p \leq 0.0001$. Experimental replicates were as follows: 2 Month Female: Wt (n=22), 5xFAD (n=25). 2 Months Male: Wt (n=14), 5xFAD (n=12). 4 Month Female: Wt (n=22), 5xFAD (n=32). 4 Month Male: Wt (n=19), 5xFAD (n=13). 8 Month Female: Wt (n=13), 5xFAD (n=23). 8 Month Male: Wt (n=21), 5xFAD (n=16). 12 Month Male: Wt (n=12), 5xFAD (n=10). 12 Month Female: Wt (n=13), 5xFAD (n=17). 13 Month Male: Wt (n=5), 5xFAD (n=5). 13 Month Female: Wt (n=9), 5xFAD (n=8). 13.5 Month Male: Wt (n=7), 5xFAD (n=6). 13.5 Month Female: Wt (n=9), 5xFAD (n=7). 14 Month Male: Wt (n=5), 5xFAD (n=5). 14 Month Female: Wt (n=9), 5xFAD (n=8).

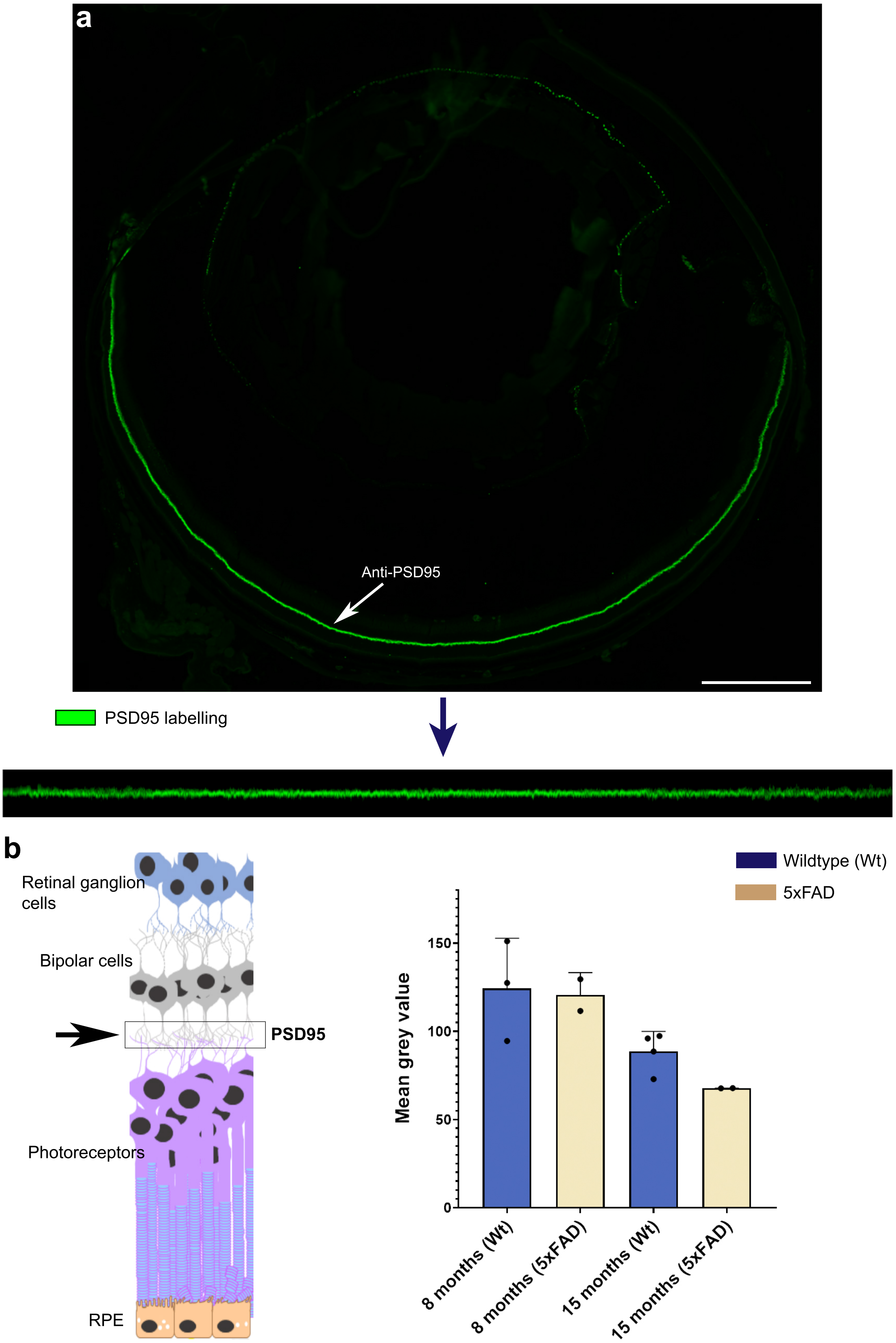


**Supplementary Figure S3: Scrutiny of postsynaptic density 95 (PSD95) labelling in 5xFAD and wildtype mouse retinae. (a)** A representative 8-bit image (x20) of an 8 month old wildtype mouse retina showing PSD95 labelling (green labelling denoted by a white arrow). Vertical arrow showing a subsequent step in the image analysis process with a region of interest (ROI) delineating PSD95 staining that has been straightened within a 300 pixel width boarder. **(b)** Schematic diagram showing PSD95 labelling of bipolar cell postsynaptic compartments that communicate with pre-synaptic photoreceptors. The mean grey value intensities of PSD95 labelling in a subset of wildtype and 5xFAD mice at 8 and 15 months were calculated using a custom-made macro. No significant differences were observed between groups, compared using either a two-tailed unpaired student t-test or Mann-Whitney U test (following tests for normality). Data is expressed as means ± SEM (standard error of mean). Scale bar in panel (a) corresponds to 500μm.

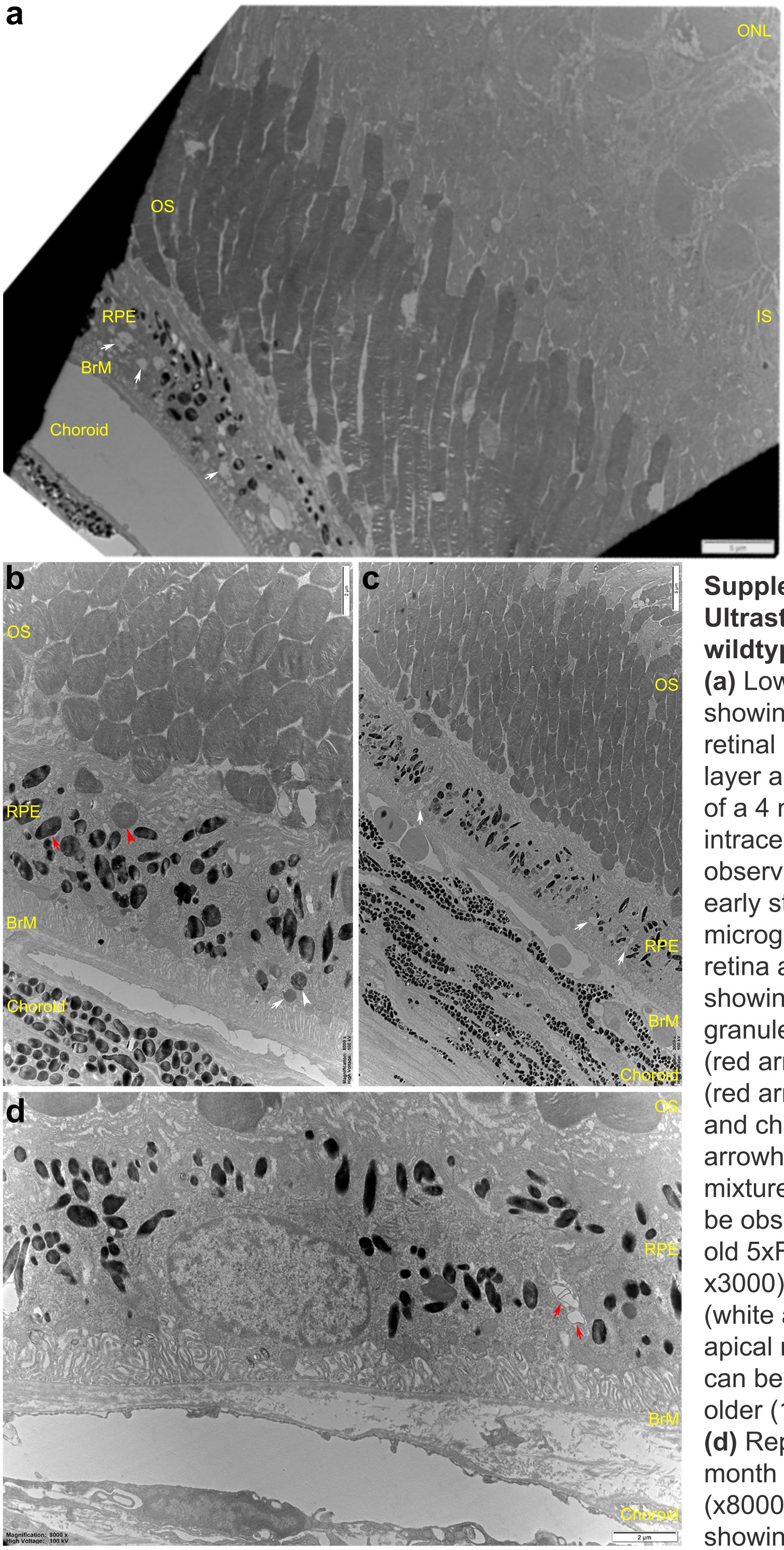


**Supplementary Figure S4: Ultrastructural analyses of 5xFAD and wildtype mouse retinae.**
**(a)** Low-powered EM micrograph (x3000) showing no obvious indication of any retinal pathology in the outer nuclear layer and in the photoreceptor IS or OS of a 4 month old 5xFAD mouse. However, intracellular RPE vacuoles can be observed (white arrows) even at this early stage. **(b)** Representative EM micrograph of a 14 month old 5xFAD retina at a higher magnification (x8000) showing a mixture of electron-dense granules including undigested POS (red arrowhead), melanolipofuscin (red arrow), lipofuscin (white arrow) and charcoal-like granules (white arrowhead) in RPE cells. **(c)** A similar mixture of electron-dense granules can be observed in RPE cells of a 14 month old 5xFAD retina (panned out image x3000) alongside vacuoles within RPE (white arrows). **(b-c)** Well-preserved apical microvilli and basolateral infolds can be observed in the RPE layer of older (14 month old) 5xFAD retinae. **(d)** Representative EM micrograph of 8 month old wildtype littermate outer retina (x8000), devoid of any pathology but showing the presence of intracellular RPE vacuoles (red arrows). Scale bars in panels a and c corresponds to 5$\mu$m, whilst those in panels b and d corresponds to 2$\mu$m. BrM: Bruch's membrane; IS: inner segments; OS: outer segments, RPE: retinal pigment epithelium.
