# Supplementary material for: A longitudinal study of the 5xFAD mouse retina delineates Amyloid beta (Aβ)-mediated retinal pathology from age-related changes
Source: Alzheimers Res Ther. 2025 Jun 19;17:136. doi: 10.1186/s13195-025-01784-w (PMC12177965; doi:10.1186/s13195-025-01784-w)
Supplement: Supplementary file 2 — Supplementary Material 2. [file 13195_2025_1784_MOESM2_ESM.pdf]

Supplementary Tables

Supplementary Table S1: Longitudinal weight measurements of 5xFAD and wildtype littermate mice. Animals were weighed at regular intervals between 2-13 months and with increased frequencies thereafter until the 14 month cull-point (wildtype, at least a total of n=14 males and females/timepoint; 5xFAD, at least a total of n=13 males and females/timepoint). The mean weight is shown for each month alongside the standard error of the mean (SEM).

Females					Males			
**Months**	**Genotype**	**Mean (g)**	**SEM**		**Months**	**Genotype**	**Mean (g)**	**SEM**
2	Wildtype	19.59	0.32		2	Wildtype	25.38	0.32
	5xFAD	18.26	0.22			5xFAD	23.62	0.32
4	Wildtype	22.64	0.34		4	Wildtype	30.14	0.36
	5xFAD	21.37	0.17			5xFAD	28.91	0.41
8	Wildtype	26.36	0.69		8	Wildtype	35	0.72
	5xFAD	23.29	0.31			5xFAD	32.72	0.80
12	Wildtype	28.59	0.88		12	Wildtype	35.63	1.47
	5xFAD	22.91	0.39			5xFAD	30.84	0.97
13	Wildtype	30.17	1.20		13	Wildtype	33.98	0.59
	5xFAD	22.91	0.39			5xFAD	29.38	0.49
13.5	Wildtype	31.63	1.317		13.5	Wildtype	34.86	0.64
	5xFAD	22.15	0.52			5xFAD	30.05	0.38
14	Wildtype	31.9	1.54		14	Wildtype	38.52	1.95
	5xFAD	20.93	0.35			5xFAD	31.27	1.02

Supplementary Table S2: PCR primers used for genotyping B6.Cg-Tg(APPSwFlLon,PSEN1*M146L*L286V)6799Vas/Mmjax mice and running conditions. The sequences of both forward (F) and reverse (R) primers used to amplify DNA fragments in real-time PCR analyses and associated thermal cycler conditions. The corresponding gene is shown alongside the expected amplicon length in base pairs (bp) and restriction digest sizes where applicable. The thermal cycler conditions and restriction digest reactions required for specific genes are also indicated. The sources of primers used in the study are also acknowledged.

Gene	Primer	Sequence (5’-3’)	Amplicon Length (bp)	Thermal Cycler Conditions	Restriction Digest	Source
OCA2	OCA2 pR262X Forward	TGTCTGTGTAGTA GTGAAGGGCTG	456	Initial Denaturation: 98ºC, 30s<br>Denaturation: 98ºC, 10s<br>Annealing: 71ºC, 20s<br>Extension:72ºC, 30s<br>Final Extension: 72ºC, 5mins	Reaction comprising 15ml DNA, 3ml 10x 3.1 Buffer, 1ml BstEII and 11ml dH_20 were run in a thermal cycler overnight at 60ºC<br><br>Wild-Type: 310 &146<br>Mutant: 310, 111 & 35<br>Heterozygote: 310, 146, 111, 35	Shoji et al Exp Anim. 2015;64(2):171-9
	OCA2 pR262X Reverse	CGTTGGCTTCCAT CAAATCC				
TYR	Tyr c. C103S Forward	GCTGAGAGTATTT GATGTAAGAAGG	841	Initial Denaturation: 98ºC, 30s<br>Denaturation: 98ºC, 10s<br>Annealing: 60ºC, 20s<br>Extension:72ºC, 30s<br>Final Extension: 72ºC, 5 mins	Reaction comprising 15ml DNA, 3ml 10x CutSmart Buffer, 1ml HypCH4III and 11ml dH_20 were run in a thermal cycler overnight at 37ºC. Enzyme heat inactivated at 80ºC for 20 mins<br><br>Wild-Type: 393, 370,78<br>Mutant: 471, 370<br>Heterozygote: 471, 393, 370, 78	N/A
	Tyr c. C103S Reverse	CAGTGAAGTTCTC ATCCCCAG				
Agouti	Agouti ASIP.A.01 Forward	GGAATTGAGAGAG GCTGTTCC	290	Initial Denaturation: 98ºC, 30s<br>Denaturation: 98ºC, 10s<br>Annealing: 60ºC, 20s<br>Extension:72ºC, 30s<br>Final Extension: 72ºC, 5mins	N/A	Zevnik et al. PLoS One. 2014;9(3):e90570
	Agouti ASIP.A.01 Reverse	ATGACTGAACTTC TGGCTCTCC				
	Agouti ASIP.na.02 Forward	GCCAGTAATTTTT CATTCTTCAG	280		N/A	
	Agouti ASIP.na.02 Reverse	ATGACTGAACTTC TGGCTCTCC				
PSEN1/ APP	Mutant Reverse: 27367	CGGGCCTCTTCG CTATTAC	Wild-Type: 216<br>Mutant: 129<br>Heterozygote: 216 & 129	Initial Denaturation: 94ºC, 3mins<br>Denaturation: 94ºC, 30s<br>Annealing: 61ºC, 30s<br>Extension:72ºC, 30s<br>Final Extension: 72ºC, 5mins	N/A	Jackson Laboratories , Protocol 31769
	Common Forward: 37598	ACCCCCATGTCAG AGTTCCT				
	Wild-Type Reverse: 37599	TATACAACCTTGG GGGATGG				

Direction	Age (Months)	Genotype																									
		Wildtype (Wt)																									
	2	-	-	-	-	-	-	-	-	-	-	-	-	-	-	-	0.422	0.4	0.433	0.425	0.422	0.428	0.467	0.419	0.55	0.453	0.386
	4	-	-	-	-	-	-	-	-	-	-	-	-	-	-	-	0.378	0.433	0.403	0.422	0.467	0.439	0.456	0.444	0.447	0.456	0.358
Clockwise (CW)	6	-	-	-	-	-	-	-	-	-	-	-	-	-	-	-	0.461	0.436	0.439	0.408	0.414	0.403	0.442	0.397	0.408	0.417	0.361
	8	-	-	-	-	-	-	-	-	-	-	-	-	-	-	-	0.397	0.4	0.383	-	0.422	0.358	0.428	0.4	0.414	0.4	0.403
	10	0.353	0.344	0.298	0.358	0.306	-	0.383	0.322	0.311	0.375	0.389	0.322	0.461	0.403	0.342	0.397	0.389	0.397	-	0.392	0.4	0.386	0.383	0.386	0.369	0.386
	12	0.431	0.325	0.3	0.394	0.422	-	0.383	0.303	0.361	0.344	0.422	0.339	0.425	0.478	0.372	0.378	0.383	0.383	-	0.339	0.375	0.389	0.353	0.383	0.389	0.375
	14	0.389	0.4	0.372	0.375	0.4	-	0.408	0.308	0.397	0.422	0.414	0.422	0.411	0.414	0.394	-	-	-	-	-	-	-	-	-	-	-
	2	-	-	-	-	-	-	-	-	-	-	-	-	-	-	-	0.456	0.428	0.422	0.419	0.342	0.392	0.422	0.392	0.4	0.467	0.383
	4	-	-	-	-	-	-	-	-	-	-	-	-	-	-	-	0.403	0.431	0.408	0.436	0.408	0.447	0.443	0.444	0.461	0.43	0.461
Counter Clockwise (CCW)	6	-	-	-	-	-	-	-	-	-	-	-	-	-	-	-	0.431	0.431	0.436	0.439	0.397	0.408	0.456	0.403	0.306	0.403	0.411
	8	-	-	-	-	-	-	-	-	-	-	-	-	-	-	-	0.392	0.389	0.397	-	0.394	0.397	0.428	0.381	0.358	0.383	0.394
	10	0.403	0.392	0.344	0.339	0.403	-	0.361	0.258	0.35	0.367	0.392	0.442	0.308	0.297	0.314	0.397	0.4	0.394	-	0.394	0.4	0.403	0.383	0.397	0.361	0.383
	12	0.561	0.333	0.383	0.356	0.431	-	0.386	0.278	0.381	0.331	0.378	0.508	0.356	0.339	0.372	0.367	0.375	0.372	-	0.367	0.383	0.397	0.319	0.372	0.372	0.372
	14	0.383	0.383	0.372	0.342	0.4	-	0.375	0.322	0.394	0.406	0.411	0.456	0.367	0.358	0.297	-	-	-	-	-	-	-	-	-	-	-
	2	-	-	-	-	-	-	-	-	-	-	-	-	-	-	-	0.4	0.425	0.45	0.411	0.481	0.439	0.417	0.406	0.422	0.386	0.378
	4	-	-	-	-	-	-	-	-	-	-	-	-	-	-	-	0.372	0.392	0.444	0.406	0.422	0.414	0.417	0.428	0.397	0.439	0.378
Up	6	-	-	-	-	-	-	-	-	-	-	-	-	-	-	-	0.408	0.381	0.411	0.408	0.406	0.397	0.436	0.403	0.389	0.417	0.389
	8	-	-	-	-	-	-	-	-	-	-	-	-	-	-	-	0.389	0.397	0.378	-	0.394	0.386	0.4	0.4	0.394	0.378	0.394
	10	0.25	0.408	0.375	0.319	0.322	-	0.322	0.289	0.269	0.339	0.556	0.478	0.381	0.378	0.411	0.397	0.394	0.4	-	0.394	0.389	0.392	0.372	0.389	0.397	0.383
	12	0.383	0.361	0.286	0.436	0.486	-	0.364	0.353	0.358	0.406	0.378	0.358	0.433	0.397	0.325	0.35	0.386	0.383	-	0.369	0.364	0.389	0.342	0.386	0.372	0.375
	14	0.389	0.397	0.336	0.356	0.392	-	0.397	0.317	0.383	0.439	0.447	0.436	0.392	0.386	0.314	-	-	-	-	-	-	-	-	-	-	-
	2	-	-	-	-	-	-	-	-	-	-	-	-	-	-	-	0.406	0.422	0.492	0.444	0.481	0.419	0.492	0.403	0.506	0.508	0.461
	4	-	-	-	-	-	-	-	-	-	-	-	-	-	-	-	0.4	0.492	0.431	0.425	0.414	0.422	0.411	0.442	0.433	0.472	0.411
Up	6	-	-	-	-	-	-	-	-	-	-	-	-	-	-	-	0.411	0.456	0.439	0.433	0.417	0.408	0.428	0.417	0.397	0.403	0.4
	8	-	-	-	-	-	-	-	-	-	-	-	-	-	-	-	0.386	0.378	0.403	-	0.386	0.381	0.408	0.397	0.386	0.397	0.392
	10	0.428	0.392	0.328	0.328	0.444	-	0.375	0.344	0.383	0.308	0.472	0.536	0.386	0.431	0.4	0.394	0.397	0.397	-	0.389	0.389	0.397	0.381	0.386	0.378	0.381
	12	0.461	0.306	0.353	0.372	0.508	-	0.353	0.444	0.375	0.369	0.392	0.556	0.433	0.456	0.483	0.364	0.378	0.372	-	0.397	0.367	0.389	0.342	0.378	0.367	0.364
	14	0.394	0.383	0.378	0.339	0.394	-	0.408	0.364	0.408	0.442	0.444	0.442	0.375	0.383	0.369	-	-	-	-	-	-	-	-	-	-	-

Direction	Age (Months)	Genotype																													
		Transgenic (5xFAD)																													
	2	-	-	-	-	-	-	-	-	-	-	-	-	-	-	-	-	0.461	0.439	0.419	0.439	0.461	0.428	0.408	0.464	0.394	0.483	0.425	0.383	0.481	0.397
	4	-	-	-	-	-	-	-	-	-	-	-	-	-	-	-	-	0.408	0.422	0.422	0.394	0.483	0.419	0.403	0.428	0.444	0.386	0.347	0.403	0.439	0.389
Clockwise (CW)	6	-	-	-	-	-	-	-	-	-	-	-	-	-	-	-	-	0.453	0.422	0.428	0.408	0.447	0.378	0.403	0.417	0.417	0.419	0.406	0.344	0.431	0.344
	8	-	-	-	-	-	-	-	-	-	-	-	-	-	-	-	-	0.336	0.342	0.372	0.406	0.411	0.4	0.389	0.394	0.406	0.392	0.325	0.4	0.411	0.328
	10	0.364	0.389	0.392	0.192	0.389	0.189		0.297	0.416	0.367	0.378	0.428	0.497	0.361	-	0.428	0.381	0.389	0.378	0.4	0.386	0.372	0.392	0.378	0.392	0.381	0.372	0.372	0.394	0.364
	12	0.325	0.414	0.397	0.361	0.394	0.383	0.286	0.086	0.358	0.278	0.325		0.375	0.422	-	-	0.325	0.331	0.347	0.383	0.386	0.339	0.336	0.361	0.367	0.339	0.306	0.333	0.3	0.314
	14	0.328	0.325	0.303	0.289	0.353	0.217	0.308		0.344	0.261	0.292		0.15	0.239	0.394	-	-	-	-	-	-	-	-	-	-	-	-	-	-	-
	2	-	-	-	-	-	-	-	-	-	-	-	-	-	-	-	-	0.333	0.4	0.414	0.447	0.406	0.414	0.469	0.492	0.422	0.556	0.419	0.425	0.356	0.511
	4	-	-	-	-	-	-	-	-	-	-	-	-	-	-	-	-	0.375	0.436	0.453	0.406	0.419	0.392	0.444	0.439	0.444	0.439	0.436	0.411	0.4	0.458
Counter Clockwise (CCW)	6	-	-	-	-	-	-	-	-	-	-	-	-	-	-	-	-	0.356	0.417	0.333	0.414	0.447	0.339	0.436	0.436	0.411	0.417	0.408	0.436	0.414	0.464
	8	-	-	-	-	-	-	-	-	-	-	-	-	-	-	-	-	0.367	0.394	0.292	0.397	0.392	0.4	0.403	0.417	0.397	0.372	0.383	0.397	0.408	0.389
	10	0.383	0.444	0.55	0.397	0.381	0.722	-	0.275	0.314	0.433	0.386	0.467	0.328	0.392	-	0.264	0.383	0.397	0.358	0.397	0.383	0.364	0.397	0.397	0.378	0.386	0.378	0.378	0.369	0.383
	12	0.333	0.2	0.422	0.411	0.403	0.506	0.392	0.2	0.378	0.444	0.342	-	0.358	0.267	-	-	0.336	0.353	0.314	0.381	0.358	0.331	0.358	0.344	0.372	0.331	0.306	0.331	0.292	0.325
	14	0.347	0.336	0.319	0.342	0.383	0.336	0.258	-	0.283	0.308	0.297	-	0.094	0.278	0.297	-	-	-	-	-	-	-	-	-	-	-	-	-	-	-
	2	-	-	-	-	-	-	-	-	-	-	-	-	-	-	-	-	0.436	0.458	0.472	0.407	0.433	0.439	0.464	0.367	0.422	0.508	0.444	0.4	0.419	0.444
	4	-	-	-	-	-	-	-	-	-	-	-	-	-	-	-	-	0.439	0.428	0.433	0.394	0.483	0.336	0.442	0.422	0.436	0.444	0.428	0.364	0.422	0.439
Up	6	-	-	-	-	-	-	-	-	-	-	-	-	-	-	-	-	0.408	0.439	0.372	0.361	0.381	0.358	0.419	0.403	0.389	0.4	0.486	0.458	0.439	0.439
	8	-	-	-	-	-	-	-	-	-	-	-	-	-	-	-	-	0.383	0.372	0.336	0.417	0.372	0.403	0.397	0.389	0.411	0.358	0.383	0.4	0.406	0.383
	10	0.422	0.364	0.319	0.3	0.586	0.311	-	0.325	0.289	0.375	0.464	0.372	0.378	0.408	-	0.525	0.389	0.4	0.378	0.4	0.389	0.375	0.392	0.389	0.364	0.386	0.372	0.378	0.383	0.381
	12	0.342	0.289	0.381	0.383	0.333	0.439	0.444	0.022	0.375	0.389	0.422	-	0.353	0.297	-	0.375	0.347	0.353	0.314	0.378	0.361	0.336	0.336	0.336	0.353	0.336	0.314	0.328	0.292	0.325
	14	0.358	0.325	0.286	0.319	0.35	0.375	0.292	-	0.258	0.292	0.314	-	0.214	0.283	0.314	-	-	-	-	-	-	-	-	-	-	-	-	-	-	-
	2	-	-	-	-	-	-	-	-	-	-	-	-	-	-	-	-	0.478	0.419	0.472	0.503	0.358	0.481	0.483	0.442	0.422	0.525	0.456	0.489	0.439	0.442
	4	-	-	-	-	-	-	-	-	-	-	-	-	-	-	-	-	0.444	0.419	0.497	0.406	0.419	0.4063	0.444	0.472	0.411	0.397	0.425	0.294	0.406	0.436
Up	6	-	-	-	-	-	-	-	-	-	-	-	-	-	-	-	-	0.403	0.417	0.378	0.378	0.397	0.358	0.422	0.436	0.408	0.408	0.411	0.428	0.425	0.361
	8	-	-	-	-	-	-	-	-	-	-	-	-	-	-	-	-	0.342	0.386	0.347	0.383	0.411	0.397	0.394	0.403	0.389	0.361	0.383	0.372	0.389	0.381
	10	0.3	0.378	0.467	0.414	0.576	0.433	-	0.208	0.403	0.375	0.3	0.431	0.447	0.325	-	0.264	0.397	0.397	0.386	0.397	0.378	0.358	0.397	0.381	0.353	0.378	0.378	0.367	0.381	0.378
	12	0.428	0.508	0.578	0.469	0.433	0.5	0.428	0.047	0.397	0.414	0.433	-	0.372	0.331	-	0.385	0.353	0.336	0.372	0.372	0.356	0.328	0.358	0.331	0.347	0.328	0.325	0.314	0.297	0.328
	14	0.325	0.331	0.294	0.336	0.325	0.386	0.253	-	0.333	0.256	0.331	-	0.111	0.256	0.369	-	-	-	-	-	-	-	-	-	-	-	-	-	-	-

Supplementray Table S3: Raw optometry data. Individual columns correspond to an individual mouse and therefore depending on the cull point may not have undergone optometry at all time points. Those that only underwent optometry at later timepoints were due to the technique not being available under the licence until mice were of that age.

Supplementary Table S4: Mean grey values of postsynaptic density 95 (PSD95) labelling in chorioretinal tissues from 8 and 15 month old 5xFAD and wildtype (Wt) littermates. The identity of tissues was anonymised prior to quantification of the PSD95 immunofluorescence labelling. Values were recorded in each of the retinal cross sections (3-4 sections per eye) and from several eyes as shown in the table. Values were averaged per animal and the mean grey value is reported for each age group in the two respective cohorts alongside their standard error of the mean (SEM).

Age (months)	Genotype	Mouse identity	Mean grey value	Average per eye	Average	SEM ±
**8**	Wt	K3	98.11	94.48	**124.30**	16.40
			103.71			
			81.62			
		K4	143.33	127.42		
			145.99			
			92.94			
		O3	178.38	151.02		
			172.56			
			102.12			
	5xFAD	K1	156.39	129.52	**120.52**	9.00
			122.32			
			117.3			
		N3	122.18	111.52		
			66.61			
			166.33			
			101.62			
**15**	Wt	I3	82.38	72.86	**88.68**	5.61
			71.97			
			64.23			
		I5	81.41	88.56		
			104.86			
			121.25			
			46.82			
		J3	84.94	95.96		
			91.7			
			111.23			
		J4	80.5	97.32		
			76.14			
			103.23			
			129.42			
	5xFAD	I4	90.5	67.74	**67.69**	0.05
			24.28			
			63.3			
			92.91			
		I7	63.1	67.64		
			25.94			
			63.96			
			117.57			

Image analysis of PSD95 labelling in 8 and 15 month old chorioretinal mouse tissues

Image analysis was carried out with Fiji software (details in methods). A Fiji macro was written to automate this process and is shown below.

```
dir1 = getDirectory("Input folder");
dir2 = getDirectory("Output folder");
dir3 = getDirectory("Results folder");
list = getFileList(dir1);

Dialog.create("Calibration & Threshold");
Dialog.addNumber("Pixel size: ", 0);
Dialog.addString("Pixel Unit: ", "nm");
Dialog.addNumber("Threshold Value: ", 0.00);
Dialog.show();
PixelSize = Dialog.getNumber();
PixelUnit = Dialog.getString();
ThresholdValue = Dialog.getNumber();

listLength = newArray();
listMean = newArray();
listStdDev = newArray();
listLabel = newArray();

for (i=0; i<list.length; i++) {

	open(dir1+list[i]);
	Title=getTitle();
	TitleA= File.nameWithoutExtension;
	ImageA = TitleA + "-1.tif";
	Duplicate = TitleA + "-1-1.tif";
	Mask = "Mask of " + TitleA + "-1-1.tif";
	CleanImage = "Result of " + ImageA;

	run("Set Scale...", "distance=1 known=1 unit=&PixelUnit");
	run("Properties...", "channels=1 slices=1 frames=1 pixel_width=&PixelSize pixel_height=&PixelSize voxel_depth=1 global");
	run("Set Measurements...", "area mean standard limit display redirect=None decimal=3");
	run("Green");
	run("8-bit");
	setTool("polyline");
	waitForUser("Outline the retina");
	roiManager("Add");
	roiManager("Select", 0);
	roiManager("Save", dir2 + "ROI " + TitleA + "-" + ".zip");
	selectWindow("ROI Manager");
	run("Close");
	selectImage(Title);
	run("Measure");
	labelValue = getResultLabel(0);
	lengthValue = getResult("Length", 0);
	listLabel = Array.concat(listLabel, labelValue);
	listLength = Array.concat(listLength, lengthValue);

	selectImage(Title);
	run("Straighten...");
	run("Duplicate...", Duplicate);
	run("Threshold...");
	setAutoThreshold("Default dark no-reset");
	waitForUser ("Set Threshold");
	run("Convert to Mask");

	setTool("wand");
	waitForUser("Identify area of particles to exclude");
	Dialog.create("Analyse particles");
	Dialog.addNumber("Area: ", 3000);
	Dialog.show();
	AreaThreshold = Dialog.getNumber();
	run("Select None");
	run("Analyze Particles...", "size=" + AreaThreshold+"-Infinity show=Masks");
	run("Divide...", "value=255.000");
	imageCalculator("Multiply create", ImageA, Mask);
	selectImage(Mask);
	close();
	selectImage(Duplicate);
	close();
	selectImage(ImageA);
	close();
	selectWindow(CleanImage);
	setAutoThreshold("Default dark no-reset");
	//run("Threshold...");
	setThreshold(ThresholdValue, 1000000000000000000000000000000.0000);
	selectWindow(CleanImage);
	run("Measure");
	MeanValue = getResult("Mean", 0);
	StdDevValue = getResult("StdDev", 0);
	listMean = Array.concat(listMean, MeanValue);
	listStdDev = Array.concat(listStdDev, StdDevValue);
	selectWindow(Title);
	run("Save");
	close();
	selectImage(CleanImage);
	saveAs("Tiff", dir2 + CleanImage);
	selectImage(CleanImage);
	close();
	close("Results");

}

Table.create("ResultsData");
Table.setColumn("Label", listLabel);
Table.setColumn("Length", listLength);
Table.setColumn("Mean", listMean);
Table.setColumn("Standard Deviation", listStdDev);

Table.save(dir3+"ResultsData.csv","ResultsData");
run("Close");

exit("ANALYSIS FINISHED");
```
